# Recent updates of the MPEXS2.1-DNA Monte Carlo code for simulations of water radiolysis under ion irradiation

**DOI:** 10.1038/s41598-025-00875-w

**Published:** 2025-05-13

**Authors:** Shogo Okada, Koichi Murakami, Tamon Kusumoto, Yoshiyuki Hirano, Katsuya Amako, Takashi Sasaki

**Affiliations:** 1 High Energy Accelerator Research Organization (KEK), 1-1, Oho, Tsukuba, Ibaraki 305-0801 Japan; 2 National Institutes for Quantum Science and Technology (QST), 4-9-1 Anagawa, Inage-ku, Chiba, 263-8555 Japan; 3 Graduate School of Medicine, Biomedical Imaging Sciences, Nagoya University, 1-1-20 Daiko-Minami, Higashi-ku, Nagoya City, Aichi Prefecture Japan

**Keywords:** Monte Carlo simulation, Water radiolysis simulation, Radiation physics, Radiation chemistry, GPGPU, Medical research, Physics, Computer science, Chemistry

## Abstract

To improve radiotherapy, especially that with ion beams such as proton and carbon ion beams, the mechanisms of interactions induced by ionizing radiation must be understood. MPEXS2.1-DNA is a Monte Carlo simulation code developed for water radiolysis studies and DNA damage simulations that uses GPU devices for fast computation. However, the original chemistry model in MPEXS2.1-DNA did not include detailed chemical reactions for reactive oxygen species (ROS), e.g., O^•-^, O_2_, O_2_^•-^, HO_2_^•^, HO_2_^-^. In the present study, drawing the former work on the step-by-step (SBS) model for the RITRACKS code, we implemented an alternative SBS model into MPEXS2.1-DNA to increase the capabilities and computational speed of water radiolysis simulations under ion irradiation. This model is based on the theory of Green’s function of the diffusion equation (GFDE-SBS). Also, we implemented multiple ionization processes which enhance ROS generation under high-LET irradiation. We compared the simulation results obtained by GFDE-SBS with experimental data from previous studies. The validation results demonstrated that the GFDE-SBS model accurately reproduced the measured radiation chemical yields of major species, such as hydroxyl radicals and hydrogen peroxide. Furthermore, the computational speed of GFDE-SBS was increased approximately ten times faster than the original model due to the changes in time stepping. Additionally, simulations using a Fricke dosimeter confirmed that this model is reliable for long-term simulations over seconds. These improvements enable simulations of radiation interactions and can help in the study of DNA damage mechanisms.

## Introduction

The number of patients receiving radiotherapy for cancer is monotonically increasing annually. Radiotherapy using ion beams (e.g., proton and C ion beams) has substantial advantages in terms of treatment effectiveness while minimizing the influence on healthy tissues surrounding tumors. To further improve radiotherapy and develop more reliable treatments, the mechanisms of interactions induced by ionizing radiation must be elucidated. In particular, the indirect action of water radiolysis species can damage DNA^1^. In an indirect action mechanism, hydroxyl radicals absorb hydrogen from the DNA backbone, converting DNA into a radical and subsequently causing strand breakage^2^. The contribution of indirect action to cell survival under X-ray irradiation was estimated to be approximately 80%^3^. Therefore, calculating the production, diffusion and reactions of water radiolysis species is essential for accurately estimating indirect action.

MPEXS2.1^4,5^ is a dose calculation code based on the Monte Carlo method that is used for medical applications, and the computation time is reduced through ultraparallel processing on NVIDIA GPU devices. The core algorithm and associated physics data, including electromagnetic, hadronic, and neutron physics data, were ported from the Geant4 simulation toolkit^6–8^. Specialized packages have been developed for the MPEXS2 series for various purposes, including X-ray and proton therapies. Among them, MPEXS2.1-DNA^9–11^ was designed to obtain global views of radiation-induced effects over a wide time scale from the physical stage (10^–3^ ps ~ 1 ps) to the biological stage (> 1 s) via the chemical stage (1 ps ~ 1 s). The behavior of typical species generated by the passage of charged particles in liquid water, such as hydroxyl radicals, hydrated electrons, and hydrogen peroxide, can be tracked via a step-by-step approach with dynamic time stepping. MPEXS2.1-DNA reproduces the experimental data under irradiation with low-linear energy transfer (LET) particles such as electrons reasonably well, as described in^11^. However, the conventional chemistry model in MPEXS2.1-DNA has not yet been optimized for accurate calculation of the radiation chemical yields of water radiolysis species under ion irradiation, such as under carbon beam irradiation. For example, the chemical reactions for reactive oxygen species (ROS), e.g., O^•-^, O_2_, O_2_^•-^, HO_2_^•^, and HO_2_^-^, are not considered. As indirect actions have been reported to greatly contribute to biological effects even under ion irradiation^1^, the implementation of missing chemical reactions is crucial for properly understanding phenomena under ionizing radiation.

In this study, MPEXS2.1-DNA was updated to increase the accuracy and computational speed of simulations of water radiolysis under ion irradiation, drawing on insights from the Monte Carlo simulation code RITRACKS (Relativistic Ion Tracks)^12–15^. RITRACKS handles numerous chemical reactions, including those involving ROS, improving simulation accuracy. It also accounts for reactions with dissolved molecules, enabling simulations of radical scavengers or oxygen effects. Additionally, its use of fixed-time steps for molecular transport reduces computation time. We also implemented multiple ionization as an additional physical process, guided by the findings of previous studies^16,17^, which demonstrated that multiple ionization plays an important role in ROS generation in high-LET region (> 200 keV/μm).

To validate our updated chemistry model, we performed simulations and compared the results with experimental data from previous studies. Specifically, we calculated the time evolution of radiation chemical yields for typical water radiolysis species, such as hydrated electrons and hydroxyl radicals, along with their LET dependencies, and compared these with the experimental measurements. The results show good agreement with the measured data. Furthermore, we confirmed the model’s applicability over extended timescales of up to several tens of seconds through Fricke dosimeter simulations. Furthermore, the updated model achieves a computational performance improvement of approximately tenfold compared to the existing model.

## Materials and methods

### MPEXS2.1-DNA

MPEXS2.1-DNA^9–11^ is a Monte Carlo simulation code run on NIVIDA GPU devices. It is based on Geant4-DNA^18–20^ version 10.7 Patch-4, which is an extension package of the Geant4 simulation toolkit^6–8^ for simulating track structures and water radiolysis at the subcellar scale. A step-by-step Brownian dynamic model for chemical processes^21^, including physics models for Geant4-DNA, has been ported using the CUDA language to reengineer data structures to make them suitable for GPU processing. This chemistry model applies the dynamic time-stepping method, adjusting a time step length depending on the distribution of molecular species at every iteration. It allows the time step length to be extended within the range where it ensures, with a 95% confidence interval, that the diffusing species will not react with a neighboring one. The minimum time step method and the Brownian Bridge technique^21^ are applied to limit the number of time steps. The minimum time step method imposes a lower bound on the time step length, which is fixed to 1 ps as a default setting. The value of the minimum step can also be increased as a function of simulation time following the proposal by the PARTRAC code^22^ to help performance improvement. The Brownian Bridge technique computes the probability of encounter during their minimum time step and thus compensates for missed reactions. Each species is transported using a time step determined in the manner mentioned above at every iteration. Motion directions are randomly chosen with a Gaussian distribution. Pairs of species make reactions if their separation distance is closer than the reaction radius derived by the Smoluchowski theory^21^. MPEXS2.1-DNA can accurately reproduce Geant4-DNA simulation results with drastic performance gains^9–11^. For water radiolysis simulations with the existing chemistry model developed in the previous study^11^, one GPU device is equivalent to 7,600 CPU cores in terms of computational power. Details are provided in a supplementary document (see Section S1).

### Implementation of an alternative step-by-step model for chemistry simulations and of multiple ionization processes in the MPEXS2.1-DNA framework

RITRACKS^12–15^ is a water radiolysis simulation code that uses a stochastic method. The RITRACKS code was written in C +  + so that simulations can be run on CPU devices. A step-by-step (SBS) approach is applied to track the behavior of each molecular species generated by the passage of charged particles in liquid water and obtain precise information on molecular positions over time. Molecular species are transported according to Gaussian random numbers while the time step is advanced, and the simulation time is discretized with a fixed step number. Although the fixed time-stepping approach can be expected to reduce computation time, the choice of these fixed-time steps may lead to a missing reaction during diffusions. Therefore, the possibility of a reaction is assessed at each time step. A chemical reaction occurring with a nearby species during diffusion is sampled using the probability calculated based on the theory of Green’s function of the diffusion equation (GFDE), in which electrostatic forces among charged molecules and spin statistics effects are considered. In addition, “background reactions,” i.e., chemical reactions with molecular species homogeneously distributed in a solution, are handled as first-order or pseudo-first-order reactions. This is an equation-based model that follows an exponential law for reaction probabilities to avoid representing dissolved species as point-like objects and thus reduce memory consumption. Thanks to the capability of background reactions, we can simulate the system where scavenging reactions become dominant at a long timescale, e.g., seen in Fricke dosimeter. The detailed theoretical background of this RITRACKS SBS model is mentioned elsewhere^12–15^.

Drawing upon the SBS model on RITRACKS described in the previous studies^12–15^, we implement an alternative SBS model based on the GFDE theory in MPEXS2.1-DNA using the CUDA language. Hereafter, we refer to this model as “GFDE-SBS.” Ultraparallel processing on GPU devices can enhance the computing performance of chemistry simulations. Regarding the parameters for chemical reactions, we employ the parameter set of the TRACIRT code^23^ because it is the most extensive parameter set available. We refer to the diffusion coefficients and radii of molecular species, including ROS^23^. These chemical parameters are summarized in a supplementary document (see Section S2). The other parameters, such as the reaction radii, reaction probabilities, and scavenging powers, are automatically calculated based on these inputs.

The previous studies^16,17^ highlighted the importance of multiple ionization in enhancing ROS generation under high-LET particle irradiation, such as carbon ions, as demonstrated by their Monte Carlo simulations. Based on these findings, we implement multiple ionization as an additional physical process for liquid water in MPEXS2.1-DNA. Double, triple, and quadruple ionizations are considered for protons (^1^H^+^), alpha particles (^4^He^2+^), and carbon ions (^12^C^6+^). The cross-sectional values are scaled based on the existing cross-sectional data of single ionization implemented in MPEXS2.1-DNA using the adjustment parameter introduced in the simulation study by Meesungnoen and Jay-Gerin^16^. The energy threshold for inducing multiple ionization, as given in the previous study^16^, is also configured for each process. The total energy in a collision between a charged particle and a water molecule must exceed this threshold for multiple electrons to be emitted from the orbitals of an H_2_O molecule. After multiple electron emission from H_2_O orbitals, the remaining molecule is treated as a multiple-ionized water ion (H_2_O^n+^, n = 2, 3, or 4). This ion dissociates into other chemical products, including an oxygen atom in a triplet P state, O(^3^P). Additional dissociative decay chains for multiple-ionized water ions are implemented^16,17^. Details are provided in a supplementary document (see Section S3).

### Simulation of radiation chemical yields for comparison with measured data

To test the accuracy of our GFDE-SBS model, we conducted simulations for comparison with theoretical calculation results and experimental data. A cubic phantom, 100 × 100 × 100 µm^3^, filled with deoxygenated water is irradiated by each primary particle, including electrons, protons (^1^H^+^), alpha particles (^4^He^2+^), and carbon ions (^12^C^6+^). The chemical reactions and diffusion of the generated molecular species are simulated by GFDE-SBS. We obtain radiation chemical yields, known as “G values,” expressed as the number of entities formed (or lost) per unit energy (traditionally 100 eV) for each molecular species. For electrons, the mono-energy of 750 keV is set to obtain the time evolution of G values for hydroxyl radicals, hydrated electrons, hydrogen peroxides, hydrogen molecules, and OH^-^ anions. For ^1^H^+^, ^4^He^2+^, and ^12^C^6+^, the incident energy is varied in the range of 0.5 MeV/u – 100 MeV/u to estimate the LET dependence of the radiation chemical yields of the molecule species. Radiation chemical yields are known to be dominated by the lower stopping power component of the track segments^24^. Thus, a small segment of the entire physical track is typically used to calculate the yields of each molecular species in water radiolysis simulations^25^, which is also beneficial to simulating many tracks in maintaining reasonable computing time. The present study applies energy thresholds against primary particles for each case: 75 keV for electrons and 10 keV for ^1^H^+^, ^4^He^2+^, and ^12^C^6+^. When the total energy loss of a primary particle exceeds the threshold, the transport of primary particles is stopped in the simulation. The source of the primary particles is located at the center of the phantom. The physical processes described in^11^ are implemented: elastic scattering, excitation, ionization, vibrational excitation, and dissociative electron attachment are for electrons; excitation, ionization, and charge exchange processes are for protons and alpha particles; regarding carbon ions, we apply ionization. In addition, multiple ionization processes are applied for ^1^H^+^, ^4^He^2+^, and ^12^C^6+^ ion irradiation, leading to the enhancement of ROS generations. The behavior of each molecule is tracked until one microsecond after irradiation. We set the log-scaled time steps following the suggestion about the number of time steps mentioned in^12^, in which the step number is fixed to 40 per order of magnitude. Thus, up to one microsecond of simulation time is discretized into 240 steps. The smallest and largest timesteps are computed to 0.059 picoseconds and 55.71 ns, respectively.

In this validation study of our GFDE-SBS model, we calculate the time evolution for molecular species, including e_aq_^-^, ^•^OH, H_2_O_2_, H_2_, and OH^-^, for electron and ion irradiation and compare them with the theoretical calculations^26–29^ and experimental data^30–41^. We also assess the LET dependency for each species mentioned above under ^1^H^+^, ^4^He^2+^, and ^12^C^6+^ ion irradiation, along with comparing with the measured data^42–51^. The number of simulated histories is 50,000 for each case.

### Fricke dosimeter simulation for validation of long-term simulations

To validate the capability of GFDE-SBS for long-timescale simulations, where chemical reactions involving scavengers become dominant, ranging from several tens to hundreds of seconds, a Fricke dosimeter simulation is performed with reference to the simulation conditions in^13,52–54^. A Fricke dosimeter consists of an aerated solution of ferrous sulfate (FeSO_4_) and sulfuric acid (H_2_SO_4_) diluted in water. Fe^2+^ and Fe^3+^ oxidation is generated by the passage of charged particles, leading to a G value of Fe^3+^,　G(Fe^3+^) = 15.6 [species/100 eV]^13^. The scavenging capacity of the Fenton reaction, which produces Fe^3+^, is 0.26 [s^-1^]. Thus, we must simulate up to ten seconds for this reaction to be complete.

The simulation setup consists of a homogeneous cubic water phantom with 6 cm sides irradiated with 100 MeV protons. The proton source is positioned at the center of the water phantom. The stopping condition for the transport of primary protons is that the energy absorbed by the phantom exceeds the threshold of 10 keV. The physical processes described in the Section II.3 are applied. The phantom contains FeSO_4_, H_2_SO_4,_ and O_2_ solutes. The concentrations are set to 400 mM for H_2_SO_4_, 5 mM for FeSO_4,_ and 250 µM for O_2_. The chemical reactions involving these dissolved molecules are handled as pseudo-first-order reactions in GFDE-SBS. Additional chemical reactions related to the Fricke solution listed in^13,52–54^, which are summarized in the supplementary document (see Section S4), are considered. The effect of acidity induced by H_2_SO_4_ should be considered, especially at high concentrations of H_3_O^+^. We implement this effect in our simulation platform, in which corrected values of reaction rate constants for chemical reactions among ions are automatically calculated, following the detailed procedure reported elsewhere^13,52,53^. To ensure the ability of a long timescale simulation in GFDE-SBS, we calculate the time evolution of the G value of Fe^3+^ and check whether the calculated G(Fe^3+^) at 100 s after irradiation reaches the reported value of 15.6 [species/100 eV]. In addition, we also obtain the LET dependency of G(Fe^3+^) and compare it with the experimental results^55–60^. Similar to water radiolysis simulations described in the previous section, we set the log-scaled timesteps, the step number to be fixed to 40 per order of magnitude. Up to 100 s are separated into 560 steps; thus, the smallest and largest timesteps are 0.059 picoseconds and 5.58 s, respectively. The number of simulated histories is set to 50,000.

## Results and discussion

### Comparison of radiation chemical yields simulated by GFDE-SBS with previously simulated and measured data

#### Time profiles of radiation chemical yields under electron irradiation

**Fig. 1:**
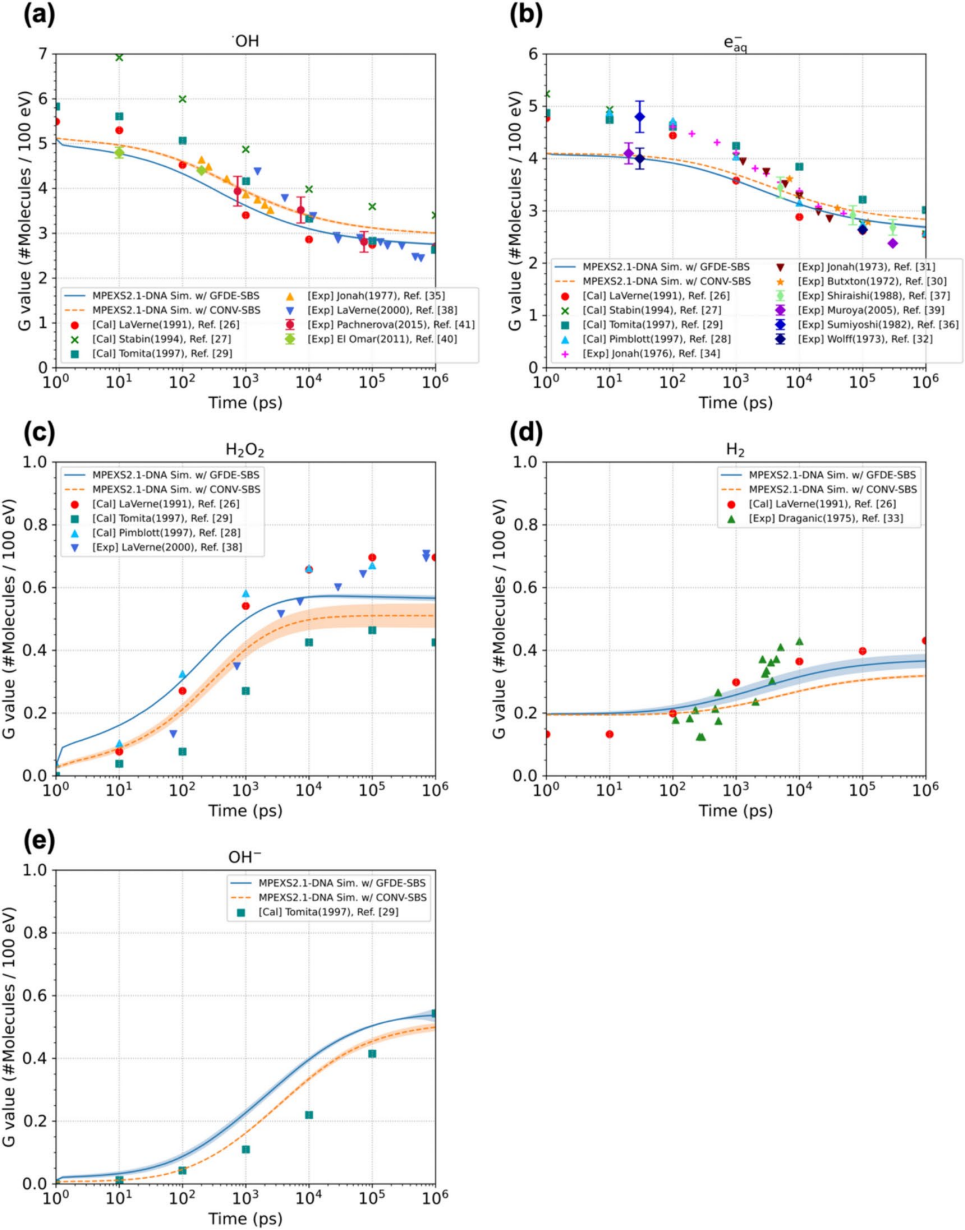
Time dependence of the G values of (**a**) hydroxyl radicals, (**b**) hydrated electrons, (**c**) hydrogen peroxide, (**d**) hydrogen molecules, and (**e**) OH^-^ anions under 750 keV electron irradiation. The solid and dashed lines are the simulation results obtained using the GFDE-SBS and CONV-SBS approaches, respectively. The symbols represent theoretical calculation^26–29^ and experimental^30–41^ data. The shade region in each panel shows the standard deviation of the calculated G value for each species. The standard deviation of hydroxyl radicals and hydrated electrons are less than 1%; thus, they are invisible.

To assess whether the GFDE-SBS model could accurately simulate water radiolysis, the time dependences of G values of typical water radiolysis species, including hydroxyl radicals (^•^OH), hydrated electrons (e_aq_^-^), hydrogen peroxide (H_2_O_2_), hydrogen molecules (H_2_), and OH anions (OH^-^), under irradiation with 750 keV electrons were examined (Fig. 1). The solid lines represent the G values calculated by GFDE-SBS. The dashed lines represent the G-values calculated by the conventional SBS model mentioned in Section II.1 (hereafter called "CONV-SBS"). The symbols represent theoretical calculation^26–29^ and measurement^30–41^ data from other studies. Here, due to the lack of reaction parameters of ROS in the default settings for CONV-SBS, the simulation of CONV-SBS applies the same parameter sets of molecular species and chemical reactions as GFDE-SBS (see Section S2 of the supplementary document). However, CONV-SBS does not have the capabilities of electrostatic forces, spin effects, and background reactions, so we cannot consider these three effects; therefore some differences in the time evolution of G values are observed compared with GFDE-SBS, as mentioned later.

The present results (GFDE-SBS) can reasonably reproduce the published data trends, including other Monte Carlo results^25,61^, for each molecular species. Compared with CONV-SBS, there are differences in the G values, mainly caused by missing the three effects mentioned above in CONV-SBS. Actually, when the calculated G values by CONV-SBS were compared with those by GFDE-SBS without the three effects, we confirmed that the differences in G values between the two models got closer. Still, depending on molecular species, specifically hydroxyl radicals and hydrogen peroxides, we observed some differences in G values, which could be caused by the differences in the reaction theory applied in each model: the combination of the Smoluchowski theory and the Brownian Bridge technique for CONV-SBS while the GFDE theory for GFDE-SBS. Details are described in Section S5 in the supplementary document.

In GFDE-SBS, we investigated the contribution of these three effects, including electrostatic forces among charged molecules, background reactions, and spin effect, to the time evolution of radiation chemical yields, which are mentioned in Section S6 of the supplementary document. Regarding hydroxyl radicals, hydrated electrons, and hydrogen peroxides, we have not confirmed significant differences induced by each effect in their G values. For hydrogen radicals, due to the spin effect, only the singlet configuration of their combined spins allows the reaction to occur, which affects the approximately 30% increase in yields of hydrogen molecules. For OH^-^ anions, the effects of electrostatic forces among nearby charged molecular species increase their yields by approximately 10%. Additionally, the background reactions originating from H_3_O^+^ and OH^-^ produced by the self-dissociation of H_2_O molecules impact the G values of OH^-^ anions. Its radiation chemical yields decrease at approximately 200 ns later due to the chemical reactions with dissolved H_3_O^+^ (OH^-^ + H_3_O^+^ → 2H_2_O). This effect is captured as the standard deviation of G(OH^-^) is promptly broadened at several hundred nanoseconds later, as shown in Fig. 1e.

#### Time evolution of the molecular species after carbon ion irradiation

**Fig. 2:**
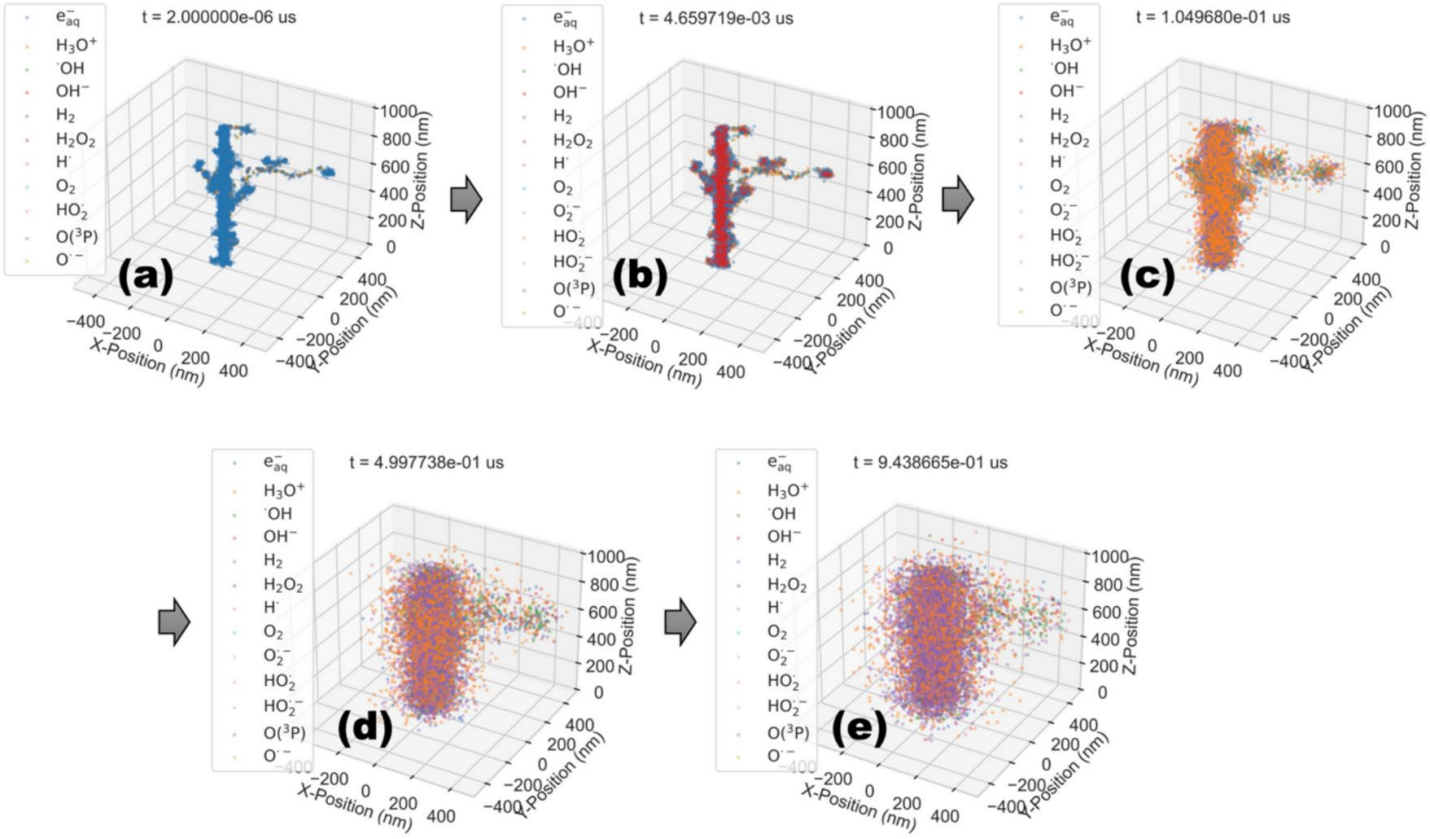
Time evolution of molecular species, simulated by the GFDE-SBS model, after irradiation by carbon ions with a kinetic energy of 5 MeV/u at (**a**) t = 2.0 x 10^–6^ µs, (**b**) 0.05 µs, (**c**) 0.10 µs, (**d**) 0.50 µs, and (**e**) 0.94 µs. Change of color is due to chemical reactions.

As mentioned, our GFDE-SBS model employs an SBS approach for molecular transport so that the diffusion of each molecular species can be precisely followed. Thus, we can evaluate the 4-dimensional evolution (x, y, z positions and time) for water radiolysis species under cabon ion irradiations with 5 MeV/u (Fig. 2). Along with the trajectory of the carbon ion and secondary electrons, water radiolysis species are formed by ionization and excitation in the physical stage (Fig. 2a). As time passes, the species spread out while interacting with neighbors, as depicted in Fig. 2b, e. By integrating biological geometries, such as the DNA double helix structure, with the SBS approach, which explicitly tracks molecular motions and interactions, we could achieve more accurate predictions of damage locations on DNA molecules caused by charged particles and ROS. This integration is expected to provide a detailed understanding of the mechanisms underlying the biological effects of irradiation through in-silico studies.

#### LET dependence of the G value under ion irradiation

**Fig. 3:**
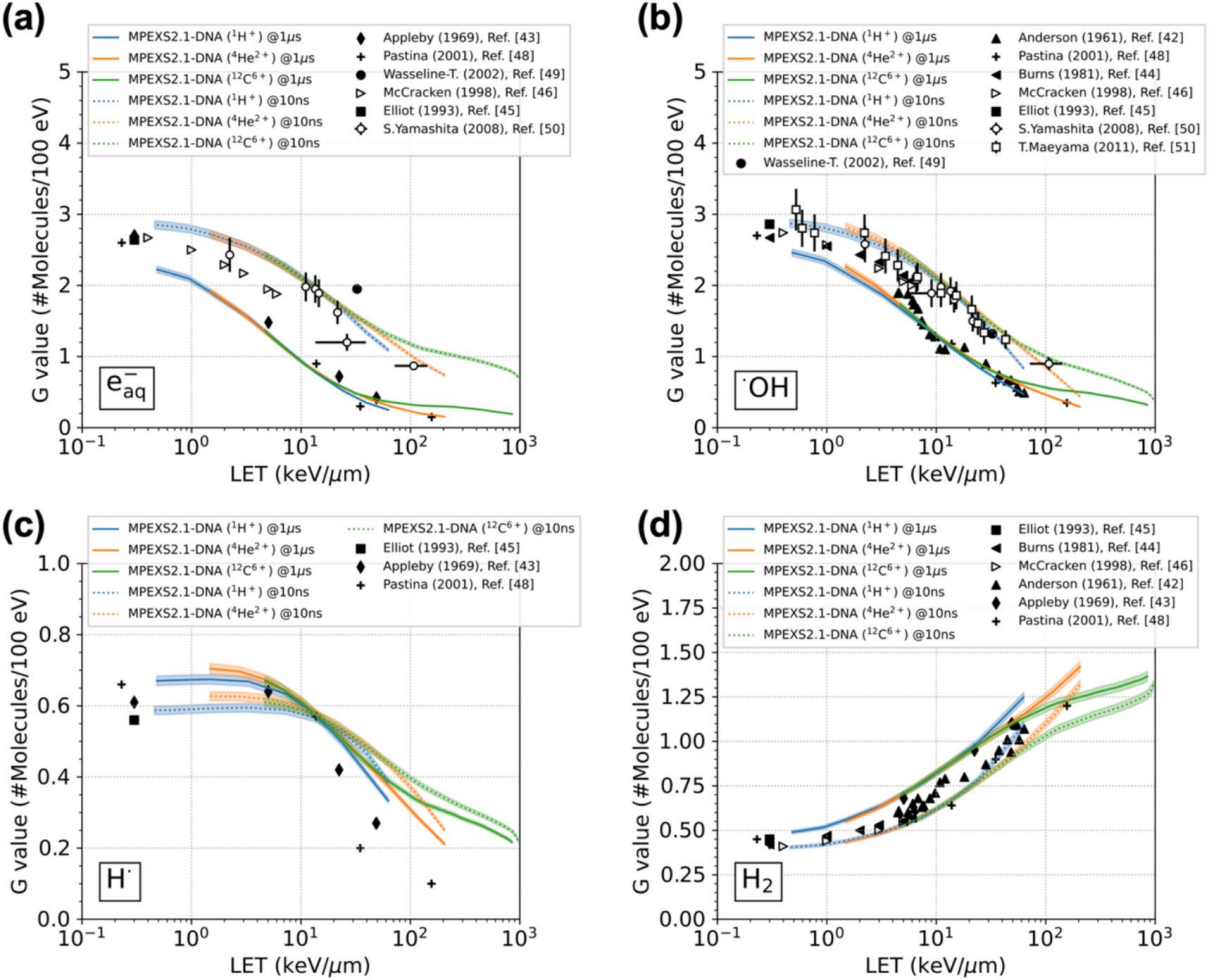
LET dependence of the chemical yields of (**a**) hydrated electrons, (**b**) hydroxyl radicals, (**c**) hydrogen radicals, and (**d**) hydrogen molecules under irradiation with protons (^1^H^+^), alpha particles (^4^He^2+^), and carbon ions (^12^C^6+^). Our simulation results obtained by GFDE-SBS for each ion are shown as solid and dashed lines of different colors. The solid lines represent the chemical yields calculated at one microsecond, whereas the dashed lines represent those calculated at ten nanoseconds after irradiation. The symbols represent experimental data^42–46,48–51^. The shaded regions represent a ± 2% range of the standard deviation of G values.

The LET dependence of the G value for typical water radiolysis products, including e_aq_^-^, ^•^OH, H^•^, and H_2_, has been reported for several decades^42–51^. However, some of the reports do not specify the time at which the G values were measured after irradiation. Experimental data are generally obtained in the presence of scavengers for specific species. Scavenging time should be adjusted from nanoseconds to microseconds scale, depending on the concentration of scavengers, to measure the time dependence of radiation chemical yields. Therefore, we compared the G values of typical water radilolysis species calculated by GFDE-SBS at ten nanoseconds and at one microsecond after irradiation by ^1^H^+^, ^4^He^2+^, and ^12^C^6+^ with experimental data (Fig. 3). The dashed lines are present results at ten nanoseconds, whereas the solid lines are our results at one microsecond. The standard deviation of the calculated G value is estimated at less than 1% for each species; therefore a ± 2% range is displayed for better visual recognition in Fig. 3. Experimental results are plotted as symbols^42–46,48–51^. The present results are reasonable to experimental results and other simulation results^16,62,63^. We ensured that the LET values calculated were consistent with the SRIM code^64^ (Section S7 of the supplementary document).

Regarding the reactions that occur during irradiation, e_aq_^-^ and ^•^OH are generated mainly from dissociative decay of excited and ionized water molecules. Each species is subsequently consumed in chemical reactions, with drastically decreasing yield over time. This is why the yield curves for e_aq_^-^ and ^•^OH at ten nanoseconds in Fig. 3 (dashed line) are higher than those at one microsecond (solid line). In contrast, H_2_ is generated through reactions involving either e_aq_^-^, ^•^OH, or H^•^, increasing their yield with time. Thus, the yield curve behavior of H_2_ in Fig. 3 is opposite to that of e_aq_^-^ and ^•^OH. Regarding H^•^, the distribution shows a shift in dominance between their production and consumption over time at approximately 15 keV/µm in LET. For example, in the case of a proton with LET of 1 keV/um, H^•^ production is dominant later so that we can observe the yield curve in the blue solid line (one microsecond) above in the blue dashed line (ten nanoseconds). On the contrary, in the proton with LET of 30 keV/um case, H^•^ consumption is dominant later; thus, the yield curve trend is the opposite. These are seen in alpha particle, and carbon ion cases as well.

As the LET increases, e_aq_^-^ and ^•^OH are generated along the ion tracks, stimulating intratrack reactions. The consumption of these species is enhanced, and their chemical yields monotonically decrease. In Section S8 of the supplementary document, the time evolution of the radiation chemical yields of e_aq_^-^ and ^•^OH are depicted under each ion irradiation, which shows rapid decreases in the G value of each species with LET increasing. In contrast, the G value of H_2_ molecules increases with increasing LET. Although the uncertainty related to the measurement time for chemical yields remains in the experimental data, our simulations reasonably capture the trends of the measured data.

#### LET dependence of the radiation chemical yields for hydrogen peroxides under ion irradiation

**Fig. 4:**
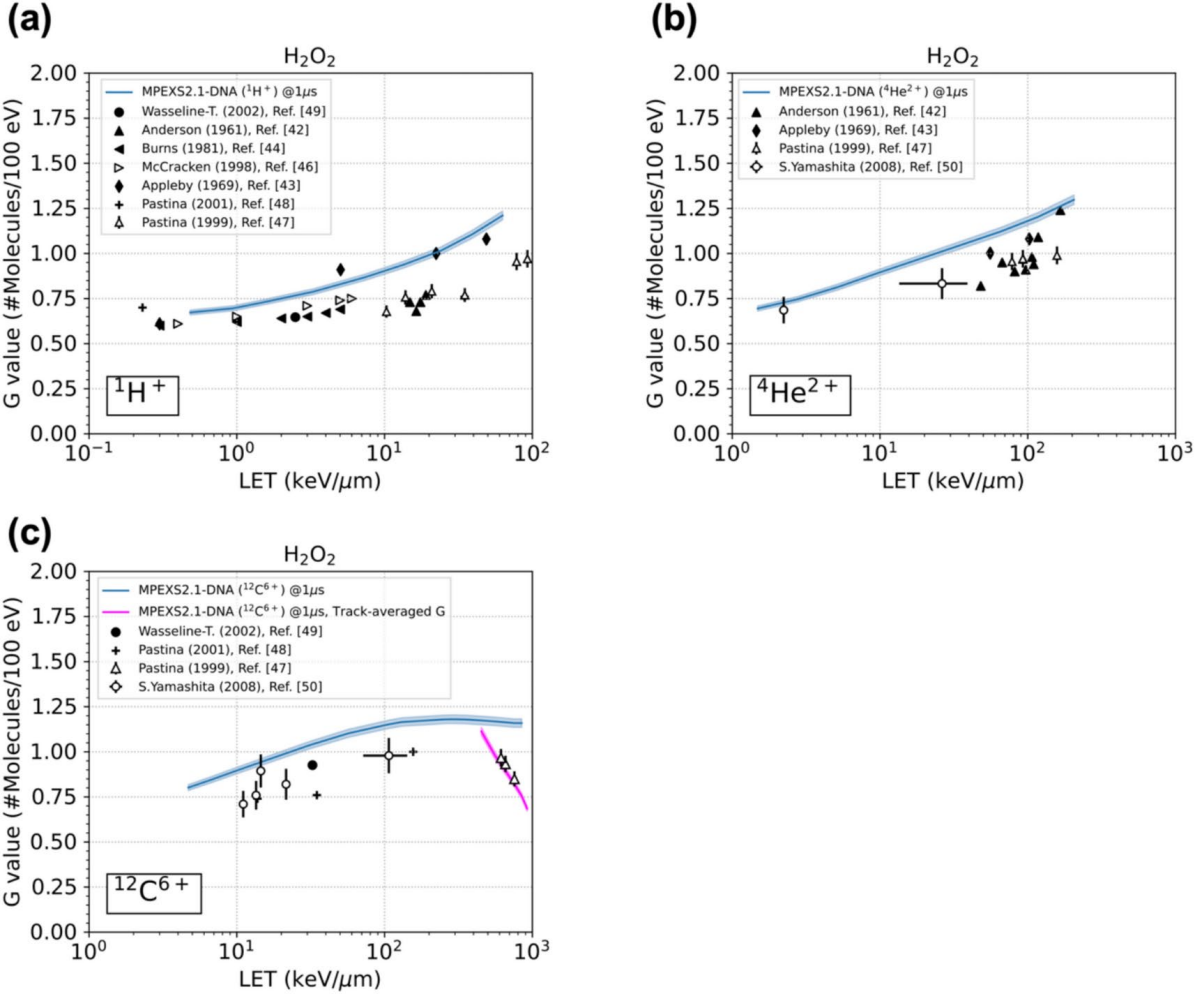
LET dependence of the chemical yield for hydrogen peroxide, G(H_2_O_2_), under irradiation with (**a**) protons, (**b**) alpha particles, and (**c**) carbon ions. The blue solid lines are our simulation results obtained by GFDE-SBS. The magenta solid line represents the reevaluation of G(H_2_O_2_) for ^12^C^6+^ as the “track-averaged G value.” The symbols represent experimental data from various studies ^42–44,46–50^. The standard deviation of G(H_2_O_2_) is less than 1% for each case; therefore, a ± 2% range is represented for better visual recognition.

Figure 4 shows G(H_2_O_2_) as a function of LET under irradiation with each ion. Our simulation results, shown as a solid blue line for each ion, were evaluated at one microsecond after irradiation. The symbols represent experimental data from previous studies^42–44,46–50^. With respect to proton and alpha particle, as shown in Fig. 4a,b, the G value of H_2_O_2_ monotonically increases with increasing LET, which agrees with the trend of the experimental data. In carbon ions, G(H_2_O_2_) reaches a maximum at a LET of approximately 200 keV/µm and then decreases. The present simulations are in agreement with experimental results and other simulations (Fig. 4c). The specific trend above 200 keV/μm is due to the contribution of the multiple ionization. Hydrogen peroxide is mainly formed by the recombination of hydroxyl radicals:

R1 $${}_{{}}^{ \bullet } \mathrm{OH} + {}_{{}}^{ \bullet } \mathrm{OH} \to \mathrm{H}_{2} \mathrm{O}_{2} \,\, \left( {k = 4.40 \times 10^9 \left[ {\left( {\mathrm{M} \cdot \mathrm{s}} \right)^{-1}} \right]} \right)$$

With increasing LET, water ions (H_2_O^n+^, n = 2, 3, 4) generated via multiple ionization dissociate into oxygen atoms in a triplet P state, O(^3^P). This process creates the following reaction paths, which lead to increases in ^•^OH consumption:

R2 $${}_{{}}^{ \bullet } \mathrm{OH} + \mathrm{O}({}_{{}}^{3} \mathrm{P}) \to \mathrm{HO}_{2}^{ \bullet } \ (k = 2.00 \times 10^{10} [(\mathrm{M} \cdot \mathrm{s})^{-1}])$$

R3 $${}_{{}}^{ \bullet } \mathrm{OH} + \mathrm{HO}_{2}^{ \bullet } \to \mathrm{O}_{2} \ (k = 9.79 \times 10^{10} [(\mathrm{M} \cdot \mathrm{s})^{-1}])$$

**Fig. 5:**
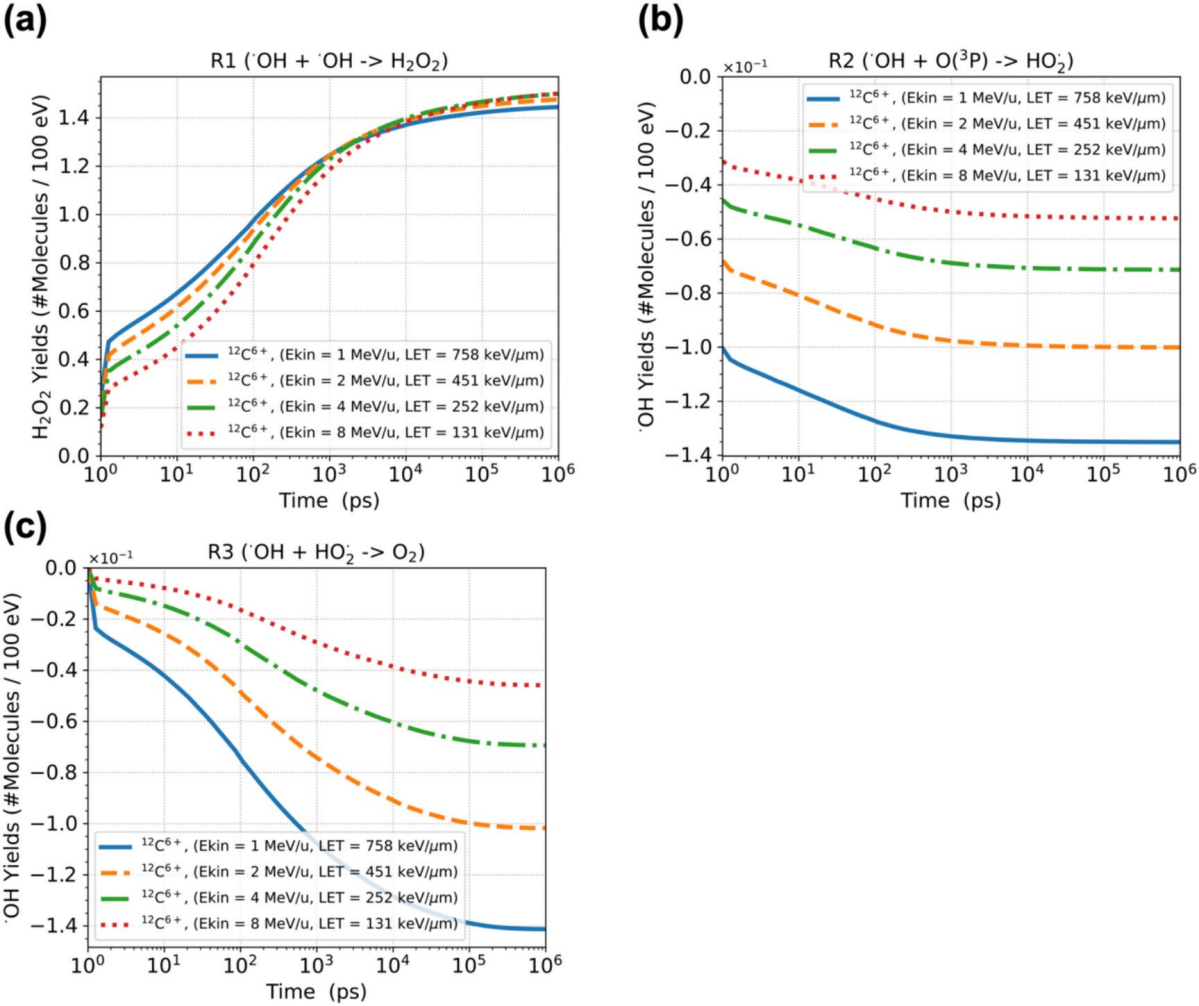
The occurrence of chemical reactions, shown as radiation chemical yields, under carbon ion irradiation with an incident energy of 1, 2, 4, and 8 MeV/u; (**a**) is the generation of hydrogen peroxides by R1, while (**b**) and (**c**) are the consumption of hydrogen peroxides by R2 and R3, respectively.

The consumption of a precursor of hydrogen peroxide, i.e., ^•^OH, through the chemical reactions of R2 and R3 as a function of time is depicted in Fig. 5, together with H_2_O_2_ generations by R1, under carbon irradiation, given incident energies of 1, 2, 4, and 8 MeV/u. As LET increases, the decreasing rate of ^•^OH by R2 and R3 are enhanced; compared to the case of 8 MeV/u (LET = 131 keV/μm) at 1 microsecond, 2.7 times higher for R2 and 3.1 times higher for R3 in 1 MeV/u (LET = 856 keV/μm), as depicted in Fig. 5b, c, respectively. This effect is captured as a decrease in the chemical yield of H_2_O_2_ at LET values greater than 200 keV/µm for carbon irradiation, as shown in Fig. 4c. In the higher LET region, where incoming carbon ions terminate their trajectories in the medium, so that the G values is treated as the “track-averaged G value” described in^47^. We reevaluated G(H_2_O_2_) for LET values greater than 200 keV/µm as the track-averaged G value, as shown by the magenta solid line in Fig. 4c. The results are in good agreement with the experimental data^47^.

#### Fricke dosimeter: Validation of long-term simulations

**Fig. 6:**
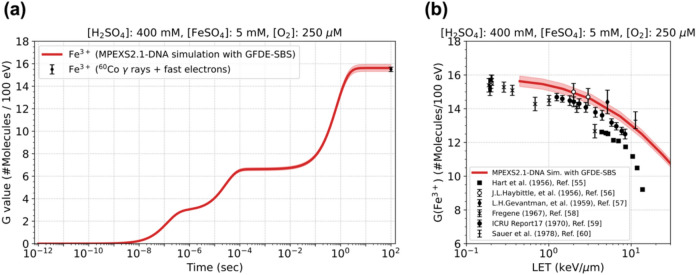
(**a**) Chemical yield of Fe^3+^ as a function of time calculated by GFDE-SBS for irradiation with 100 MeV protons up to 100 s (red solid line). The filled circle with a vertical error bar represents the reported chemical yield of Fe^3+^. (**b**) LET dependence of G(Fe^3+^). The solid red line represents the simulation results, calculated at 100 s after irradiation with protons of various incident energies. The symbols represent measured data from various studies^55–60^. A ± 2% range of the standard deviation of G(Fe^3+^) is displayed as a shaded region.

To demonstrate whether long-term simulations, where scavenging reactions become dominant, are valid by our GFDE-SBS model, we performed a Fricke dosimeter simulation. Figure 6a shows the time evolution of the chemical yields of Fe^3+^ generated by proton irradiation with kinetic energy of 100 MeV. Several tens of seconds after irradiation, the radiation chemical yields reach a plateau where G(Fe^3+^) is approximately 15.6 [species/100 eV], which agrees with the ICRU reported value of 15.6 ± 0.2 [species/100 eV]. We also studied the LET dependence of G(Fe^3+^) by varying the incident proton energy from 200 keV to 100 MeV. Figure 6b shows the calculated G(Fe^3+^) at 100 s after irradiation as a function of LET together with experimental data^55–60^. The standard deviation of calculated G(Fe^3+^) is estimated to be less than 1%; therefore, in Fig. 6, ± 2% range is depicted as a shaded region to improve visual recognition. As the LET increases, the chemical yield of Fe^3+^ decreases from 15 to 10 [species/100 eV]. Our simulation results agree with the experimental data up to 10 keV/µm. Thus, we have confirmed that our GFDE-SBS model works for simulations on a long timescale of several tens of seconds.

#### Computational performance of the GFDE-SBS model

To demonstrate the computational performance improvement of GFDE-SBS, Table 1 shows the performance metrics of GFDE-SBS compared with those of CONV-SBS for irradiation by ^1^H^+^, ^4^He^2+^, and ^12^C^6+^ with a kinetic energy of 20 MeV/u. The GPU device used in this benchmark was an NVIDIA® RTX™ 6000 Ada Generation. We measured the histories per second (HPS) score, defined as the number of histories processed per second, to evaluate the computational speed of the models. The computational performance of GFDE-SBS is more than approximately ten times faster than that of CONV-SBS for each case. The computation time is directly affected by the number of time steps. The mean numbers of time steps for each case are presented in Table 1. In the case of CONV-SBS, the mean number of time steps for each ion is approximately ten times greater than that in the case of GFDE-SBS. As described in Section II.1, a dynamic time-stepping method^21^ is applied in CONV-SBS. The time step length is automatically adjusted based on the distribution of molecular species at every iteration to guarantee that none of the molecules are closer than the reaction radius. Therefore, if many species are densely generated, the time step length should be shortened, and the computation time will become much longer with an increasing number of iterations. As molecular species tend to be densely distributed for high-LET ions such as carbons, as shown in Fig. 2, the time step length becomes shorter than that for other low-LET particles, leading to an increase in the number of steps. In contrast, in GFDE-SBS, as mentioned before, fixed time stepping is applied; the number of steps is fixed at 240 for all cases. Thus, the GFDE-SBS model can drastically reduce computation time while reasonably reproducing the experimental data, as shown in Figs. 1, 3, 4, and 6.

**Table 1:** Performance comparison between two chemistry models, CONV-SBS and GFDE-SBS, for ^1^H^+^, ^4^He^2+^, and ^12^C^6+^ irradiation with a kinetic energy of 20 MeV/u. The performance gain is defined as the HPS score of GFDE-SBS over that of CONV-SBS.

		Mean number of time steps	HPS Score
^1^H^+^ 20 MeV/u	GFDE-SBS	240 steps	777 histories/sec
	CONV-SBS	18,856 steps	58 histories/sec
	Performance gain		13.3 times
^4^He^2+^ 20 MeV/u	GFDE-SBS	240 steps	695 histories/sec
	CONV-SBS	23,744 steps	50 histories/sec
	Performance gain		13.9 times
^12^C^6+^ 20 MeV/u	GFDE-SBS	240 steps	667 histories/sec
	CONV-SBS	23,872 steps	67 histories/sec
	Performance gain		9.9 times

### Future prospects

As demonstrated in the present study, the chemical yields of molecular species under ion irradiation can be accurately determined by implementing the GFDE-SBS model, which handles chemical reactions of ROS and dissolved molecules, in the MPEXS2.1-DNA framework. In the future, by integrating biological models and DNA double helix structures into our simulation framework, the impact of indirect actions on DNA molecules induced by carbon beams, e.g., base lesions and strand breakage and repair, could be quantitatively estimated. The combination of the hydroxyl radical distribution calculation in this study and the model used in^65^ could enable us to roughly estimate the distribution of single- or double-strand breaks, as well as base and sugar damage, including lethal and reparable types of damage. Cell survival could be estimated if these types of damage were identified.

In radiation therapy, the oxygen effect, in which the radiosensitivity of living cells is decreased under hypoxic conditions, is particularly important. Calculating the number and distribution of oxygen molecules is important for estimating this effect. A previous study quantitatively assessed the increase in radiosensitivity caused by the passage of carbon ions using Geant4-DNA^66^. In this study, to reduce the computation time, a combination of the independent reaction time (IRT) method^67,68^ and numerical solutions of the diffusion equation was used to calculate changes in the oxygen concentration over a long duration (~ 1 ms). However, the IRT method trades off precise determination of molecule positions for computational speed, which limits its ability to estimate radiation-induced biological impacts with high spatial accuracy. In contrast, MPEXS2.1-DNA can efficiently track molecule locations, including damage positions, at every time step over long durations, thanks to its GFDE-SBS model, while maintaining reasonable computation times.

By calculating the chemical yields of Fe^3+^ in a Fricke solution, we confirmed that our GFDE-SBS model can perform long-term simulations up to several hundred seconds. Furthermore, our GFDE-SBS model employs a fixed time step for molecular transport, allowing approximately ten times faster simulations than CONV-SBS. These advantages would be beneficial in water radiolysis simulations under ultrahigh dose rate (UHDR) conditions because a large number of molecular species must be tracked. This required tracking, together with the continuous particle irradiation that lasts from milliseconds to seconds, leads to a much greater computational load. We expect our GFDE-SBS model to address these challenges in performing UHDR irradiation simulations.

## Conclusion

By extending the functionalities of MPEXS2.1-DNA through the inclusion of the GFDE-SBS model and multiple ionization processes, which enable treatment of chemical reactions of ROS and dissolved molecules in solution, we have increased the capabilities of water radiolysis simulations under ion irradiation. Our simulation results can reproduce the measured data reported in previous studies regarding the chemical yields of various molecular species under electron, proton, alpha particle, and carbon irradiation. Additionally, we confirmed that our GFDE-SBS model works for chemistry simulations with a long timescale above hundreds of seconds through a validation study in which the chemical yields of Fe^3+^ in Fricke solutions were calculated. Because our GFDE-SBS model employs fixed time stepping for molecular transport, more efficient chemistry simulation computations are possible. Additionally, owing to the SBS approach, we can precisely calculate the positions of each molecular species at every iteration. These advantages may be beneficial in quantitatively estimating the degree of DNA damage induced by ions and DNA repair in combination with biological models. Moreover, water radiolysis simulations under UHDR conditions, in which a vast number of molecular species need to be tracked one by one, can be accelerated to obtain insights associated with FLASH effects from in silico studies.

## Supplementary Information

Supplementary Information.

## Data Availability

The datasets generated during and analyzed during the current study are available from the corresponding author on reasonable request.
