# Supplementary material for: Recent updates of the MPEXS2.1-DNA Monte Carlo code for simulations of water radiolysis under ion irradiation
Source: Sci Rep. 2025 May 13;15:16534. doi: 10.1038/s41598-025-00875-w (PMC12075733; doi:10.1038/s41598-025-00875-w)
Supplement: Supplementary file 1 — Supplementary Information. [file 41598_2025_875_MOESM1_ESM.pdf]

Supplementary Document for "Recent updates of the MPEXS2.1-DNA Monte Carlo code for simulations of water radiolysis under ion irradiation"

Shogo Okada[1,*], Koichi Murakami[1], Tamon Kusumoto[2], Yoshiyuki Hirano[3], Katsuya Amako[1], and Takashi Sasaki[1]

1) *High Energy Accelerator Research Organization (KEK), 1-1, Oho, Tsukuba, Ibaraki, 305-0801, Japan*

2) *National Institutes for Quantum Science and Technology (QST), 4-9-1 Anagawa, Inage-ku, 263-8555, Chiba, Japan*

3) *Graduate School of Medicine, Biomedical Imaging Sciences, Nagoya University 1-1-20 Daiko-Minami, Higashi-ku, Nagoya City, Aichi Prefecture*

*Corresponding Author, Email: shogo.okada@kek.jp

S1 MPEXS2.1-DNA Performance

Figure S1 shows the time evolution of the radiochemical yields, well-known as a G-value[a], for hydroxyl radicals, hydrogen peroxides, solvated electrons, and hydrogen molecules under 750 keV electron irradiations. The solids and dashed lines are simulation results by MPEXS2.1-DNA and Geant4-DNA version 10.7 Patch-4, respectively. The other plots are measured data from various studies [6-17]. Here, we used the existing chemistry model in MPEXS2.1-DNA, referred as "CONV-SBS" in the main document. MPEXS2.1-DNA can reproduce the Geant4-DNA simulation results precisely. Also, it is in good agreement with measured data. Table S1 represents the performance gain of MPEXS2.1-DNA against Geant4-DNA simulation. Here, we measured the number of histories processed per minute for both cases. The Geant4-DNA simulation was performed with a single CPU core. On the other hand, MPEXS2.1-DNA ran around 500,000 GPU threads. The CPU and GPU devices we used are shown in Table S1. The speedup factor is defined as the throughput of MPEXS2.1-DNA over that of Geant4-DNA. MPEXS2.1-DNA is faster than 7,600 times faster than Geant4-DNA. In other words, one GPU card corresponds to the computing performance of 7,600 CPU cores in that simulation.

---

[a] The number of molecular species generated by the energy deposition of 100 eV.

Table S1. Performance comparison between the two simulation codes, including Geant4-DNA and MPEXS2.1-DNA, in the water radiolysis simulations under 750 keV electron irradiations.

	CPU/GPU	Throughput (#Histories/min.)	Speedup Factor (= MPEXS2.1-DNA / Geant4-DNA)
**Geant4-DNA ver 10.7 Patch4**	Intel® Xeon® Gold 6326 (Ice Lake)	5.7	-
**MPEXS2.1-DNA (CONV-SBS)**	NVIDIA® RTX™ 6000 Ada Generation	43,565.0	7,643

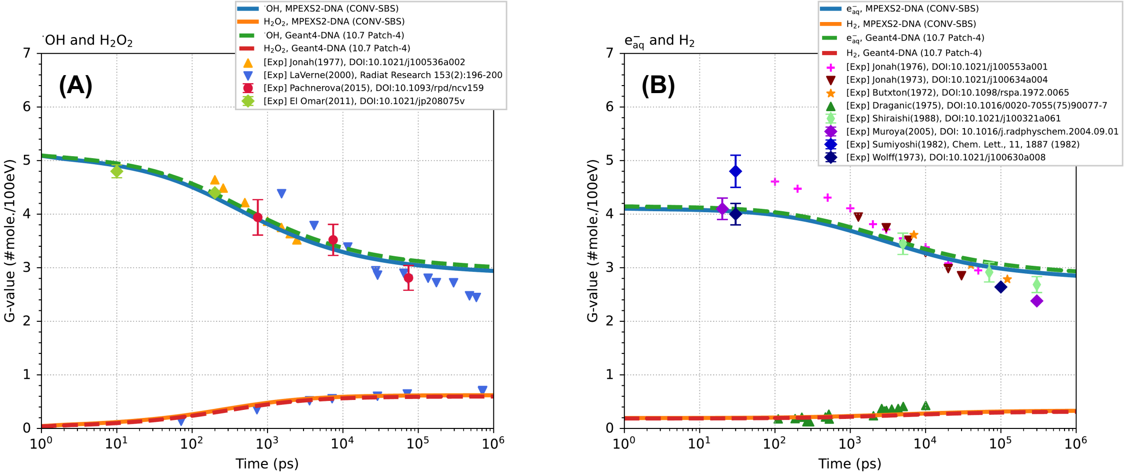

Figure S1. The time evolution of G-values of hydroxyl radicals and hydrogen peroxide in (A), solvated electrons, and hydrogen molecules in (B). The solid and dashed lines are simulation results by MPEXS2.1-DNA and Geant4-DNA ver. 10.7 patch-4, respectively. The other plots are experiment data from various studies [6-17].

S2 Implementation of the GFDE-SBS model in MPEXS2.1-DNA

Drawing upon the step-by-step (SBS) model on the RITRACKS code described in the previous studies [2,18-20], we implemented an alternative SBS model based on the theory of Green's function of the diffusion equation (GFDE) in the MPEXS2.1-DNA framework. As described in the main document, we refer to this model as "GFDE-SBS." In this section, we briefly summarize the theoretical background described in [2,18-20] and the parameter set of chemical reactions our GFDE-SBS model uses.

At the chemical stage of MPEXS2.1-DNA simulation with the GFDE-SBS model, intermolecular distances r_0 are calculated for each molecule pair distributed every time step. Then, they are compared with the reaction radius R for the corresponding reaction. If $r_0 < R$ (collision), the occurrence of chemical reactions called "contact reaction" is sampled with the probability P_{contact}, which is calculated as follows:

$$P_{\mathrm{contact}} = \frac{\exp\left[-\frac{r_c}{R}\right] - \exp\left[-\frac{r_c}{R + R_s}\right]}{\exp\left[-\frac{r_c}{R}\right] - \exp\left[-\frac{r_c}{R + R_s}\right] - \frac{k_{\mathrm{dif}}}{k_{\mathrm{act}}}\left[1 - \exp\left(-\frac{r_c}{R}\right)\right]}, \tag{1}$$

where r_c is the Onsager radius defined as the distance at which the Coulomb potential energy between two-unit charges is equal in the magnitude to $k_B T$, k_B being the Boltzmann constant and T the absolute temperature of medium. R_s is the separation distance, taken as 0.3 nm. k_{act} is an activation rate constant, which is linked to the experimental reaction rate constant k_{obs} and the diffusion rate constant k_{dif} :

$$\frac{1}{k_{\mathrm{obs}}} = \frac{1}{k_{\mathrm{dif}}} + \frac{1}{k_{\mathrm{act}}}. \tag{2}$$

It is possible to obtain k_{act} from k_{obs} and k_{dif}.

If $r_0 > R$, each molecular species of the pair will diffuse in random directions without the occurrence of contact reactions. However, they have a chance to collide and make a reaction during diffusion. The GFDE-SBS model classifies individual chemical reactions into four categories depending on the charged state of molecular species and contact reaction probabilities. The first two categories are for neutral molecules, and the latter are for charged molecules. The reaction probabilities during diffusion are defined with respect to each category, shown below.

The type-1 reactions correspond to fully (P_{contact} = 1) diffusion-controlled reactions for neutral molecules. The probability $P_{\mathrm{I}}(\Delta t|r_0)$ that a pair of molecular species with an intermolecular distance r_0 will diffuse in Δt and react is given by:

$$P_{\mathrm{I}}(\Delta t|r_0) = \frac{R}{r_0}\mathrm{Erfc}\left[\frac{r_0 - R}{4D\Delta t}\right], \tag{3}$$

where Erfc(x) is the complementary error function. *D* is the sum of the diffusion coefficients of two reactants. The experimental reaction rate constant, k_{obs}, is linked to the reaction radius, *R*, by $k_{\mathrm{obs}} = 4\pi RD$. So, in this case, reaction radius is derived by $R = k_{\mathrm{obs}}/4\pi D$.

The type-2 is a partially ($P_{\mathrm{contact}} < 1$) diffusion-controlled reaction for a neutral molecule. The reaction probability $P_{\mathrm{II}}(\Delta t|r_0)$ is defined as follows:

$$P_{\mathrm{II}}(\Delta t|r_0) = \frac{R\alpha + 1}{r_0\alpha}\left[\mathrm{Erfc}\left(\frac{r_0 - R}{\sqrt{4D\Delta t}}\right) - W\left(\frac{r_0 - R}{\sqrt{4D\Delta t}}, \alpha\sqrt{D\Delta t}\right)\right], \tag{4}$$

where $W(x, y) = \exp(2xy + y^2)\mathrm{Erfc}(x + y)$, and $\alpha = -(k_{\mathrm{act}} + 4\pi RD)/(4\pi R^2 D)$. The diffusion reaction rate constant k_{dif} is defined as $k_{\mathrm{dif}} = 4\pi DR$. In the case of type-2 reactions, *R* is taken as the sum of the radii of two reactants.

The type3 reaction is a fully ($P_{\mathrm{contact}} = 1$) diffusion-controlled reaction for a charged molecule. The reaction probability $P_{\mathrm{III}}(\Delta t|r_0)$ is given by:

$$P_{\mathrm{III}}(\Delta t|r_0) = \frac{R_{\mathrm{eff}}}{r_{\mathrm{eff}}}\mathrm{Erfc}\left[\frac{r_{\mathrm{eff}} - R_{\mathrm{eff}}}{4D\Delta t}\right], \tag{5}$$

where the natural distance scale of the radial process is given by effective distances r_{eff} and R_{eff}:

$$r_{\mathrm{eff}} = \frac{-r_c}{1 - \exp\left(\frac{r_c}{r}\right)} \quad \text{and} \quad R_{\mathrm{eff}} = \frac{-r_c}{1 - \exp\left(\frac{r_c}{R}\right)}. \tag{6}$$

The effective distances are positive values for *r* or $R > 0$. By analogy of type-1 reactions, the reaction rate constant k_{obs} can be linked to R_{eff} by $R_{\mathrm{eff}} = k_{\mathrm{obs}}/4\pi D$. When $r_c \to 0$ (no electric fields between molecules), $r_{\mathrm{eff}} \to r$ and $R_{\mathrm{eff}} \to R$, so $P_{\mathrm{III}}(\Delta t|r_0) \to P_{\mathrm{I}}(\Delta t|r_0)$.

The type4 is a partially ($P_{\mathrm{contact}} < 1$) diffusion-controlled reaction for molecular species with charged state. The reaction probability $P_{\mathrm{IV}}(\Delta t|r_0)$ is derived as follows:

$$P_{\mathrm{Iv}}(\Delta t|r_0) = \frac{R''_{\mathrm{eff}}}{r_{\mathrm{eff}}}[\mathrm{Erfc}(b) - W(b, a)], \tag{7}$$

where

$$a = \frac{4R^2\alpha}{r_c^2}\sqrt{\frac{\Delta t}{D}}\sinh^2\left(\frac{r_c}{2R}\right), \tag{8}$$

$$b = \frac{r_c}{4\sqrt{D\Delta t}}\left[\coth\left(\frac{r_c}{2r}\right) - \coth\left(\frac{r_c}{2R}\right)\right], \tag{9}$$

$$\alpha = \nu + \frac{r_c D}{R^2[1 - \exp(-r_c/R)]}, \ \nu = \frac{k_{\text{act}}}{4\pi R^2}, \tag{10}$$

$$R"_{\text{eff}} = \frac{r_c}{\exp\left(\frac{r_c}{R}\right)\left(1 + \frac{R r_c}{R^2 \nu}\right) - 1}. \tag{11}$$

In this case, the diffusion reaction rate constant k_{dif} is given by $k_{\text{dif}} = 4\pi D R_{\text{eff}}$, and the activation rate k_{act} is found by Eq. (2). When $r_c \to 0$ (no electric fields between molecules), $P_{\text{IV}}(\Delta t|r_0) \to P_{\text{II}}(\Delta t|r_0)$.

The simulation kernel switches to sample background reactions (referred as type-6 reactions in [2,18-20]) if each molecular species makes no reactions, including contact and diffusion-controlled ones (type-1 to 4) described above, at each time-stepping. As mentioned, background reactions are handled as first-order or pseud-first-order reactions involving molecular species homogeneously distributed in a solution. When species-A reacts with a dissolved species-B in a solution, assuming that the concentration of species [B] is high enough not to be changed by chemical reactions, the reaction occurrence rate of species-A should be written as:

$$\frac{\mathrm{d}[A]}{[A]} = -k[B]dt \tag{12}$$

$\mathrm{d}[A]/[A]$ can be interpreted as the probability that species A reacts with dissolved species B. The coefficient $k[B]$ is well-known as a scavenger power, which has a unit of s^{-1}, and is regarded as a lifetime of species A. Thus, the reaction probability for species A involving a dissolved species B during time-stepping Δt is given by:

$$P = 1 - \exp\left(-k[B]\Delta t\right) \tag{13}$$

We sample background reactions using the probability calculated by Eq.(13).

As mentioned in the main document, our GFDE-SBS model applies the parameter set for chemical reactions described in [21]. Molecular species along with their diffusion coefficients and radii are summarized in Table S2. Chemical reactions are shown in Table S3.

Table S2. Molecular species handled in the GFDE-SBS model of MPEXS2.1-DNA along with their diffusion coefficients and radii.

Species	Diffusion Coefficient [×10^{-9} m^2/s]	Radius [nm]	Species	Diffusion Coefficient [×10^{-9} m^2/s]	Radius [nm]
e^-_{aq}	4.9	0.5	$O_2^{\bullet-}$	1.75	0.22
H_3O^+	9.46	0.25	$HO_2^{\bullet}$	2.3	0.21
$H^{\bullet}$	7.0	0.19	HO_2^-	1.4	0.25
$^{\bullet}OH$	2.2	0.22	$O(^3P)$	2.0	0.2
H_2O_2	2.3	0.21	$O^{\bullet-}$	2.0	0.25
H_2	4.8	0.14	$O_3^{\bullet-}$	2.0	0.2
OH^-	5.3	0.33	O_3	2.0	2.0
O_2	2.4	0.17			

S3 Multiple Ionization

We summarize the parameters related to multiple ionization processes for protons ($^1H^+$), alpha particles ($^4He^{2+}$), and carbon ions ($^{12}C^{6+}$) ions. The cross-section values for double-, triple-, and quadruple-ionization are scaled based on the existing cross-section data of single ionization[b] using an adjustment parameter (α). This parameter is defined as the ratio of cross-section values for double-ionization over ones of single-ionization ($\alpha = \sigma_{di}/\sigma_{si}$). It is introduced in the simulation study performed by Meesungnoen and Jay-Gerin[22] to reproduce the summation of the measured chemical yield of $HO_2^{\bullet}$ and $O_2^{\bullet-}$, G($HO_2^{\bullet}$ + $O_2^{\bullet-}$), which multiple-ionization directly affects. The authors determined the α parameters as a function of ion energy per nucleon for each particle, shown in Figure S2. Details are mentioned in the previous studies[22]. We also set the energy threshold for each process. The total energy in a collision between a charged particle and a water molecule needs to exceed this threshold to emit multiple electrons from the orbits of an H_2O molecule. The cross-section and the energy threshold for each process are described in Table S4. Additional dissociative decay chains related to multiple-ionized water ions (H_2O^{n+}, n = 2, 3, 4) implemented into the MPEXS2.1-DNA framework shown in Table S5.

---

b The single-ionization process of MPEXS2.1-DNA corresponds to the G4DNARuddIonisationExtendedModel class for Geant4 version 10.7 Patch-4 release.

Table S3. Chemical reactions and their reaction rate constants (k_{obs}) considered in the GFDE-SBS model in MPEXS2.1-DNA. For first-order reactions, indicated by the symbol *, the value of k_{obs} is given in s^{-1}.

Reaction	Type	k_{obs} [/(M•s)]	Reaction	Type	k_{obs} [/(M•s)]
$H^\bullet + H^\bullet \rightarrow H_2$	1	5.03×10^{9}	$e^-_{aq} + HO^\bullet_2 \rightarrow HO^-_2$	2	1.28×10^{10}
$H^\bullet + e^-_{aq} \rightarrow H_2 + OH^-$	1	2.65×10^{10}	$OH^- + HO^\bullet_2 \rightarrow O^{\bullet-}_2 + H_2O$	2, 6	1.27×10^{10}
$H^\bullet + O(^3P) \rightarrow OH^\bullet$	1	2.00×10^{10}	$OH^- + O(^3P) \rightarrow HO^-_2$	2, 6	4.20×10^{8}
$H^\bullet + O^{\bullet-} \rightarrow OH^-$	1	2.00×10^{10}	$O_2 + O(^3P) \rightarrow O_3$	2	4.00×10^{9}
${}^\bullet OH + O(^3P) \rightarrow HO^\bullet_2$	1	2.00×10^{10}	$O_2 + O^{\bullet-} \rightarrow O^{\bullet-}_3$	2	3.70×10^{9}
$HO^\bullet_2 + O(^3P) \rightarrow O_2 + {}^\bullet OH$	1	2.00×10^{10}	$HO^\bullet_2 + HO^\bullet_2 \rightarrow H_2O_2 + O_2$	2	6.75×10^{5}
$O(^3P) + O(^3P) \rightarrow O_2$	1	2.20×10^{10}	$HO^\bullet_2 + O^{\bullet-}_2 \rightarrow HO^-_2 + O_2$	2	7.48×10^{7}
$H^\bullet + {}^\bullet OH \rightarrow H_2O$	2	1.44×10^{10}	$HO^-_2 + O(^3P) \rightarrow O^{\bullet-}_2 + {}^\bullet OH$	2	5.30×10^{9}
$H^\bullet + H_2O_2 \rightarrow H_2O + {}^\bullet OH$	2	5.18×10^{7}	$e^-_{aq} + e^-_{aq} \rightarrow H_2 + 2OH^-$	3	6.36×10^{9}
$H^\bullet + OH^- \rightarrow H_2O + e^-_{aq}$	2, 6	2.51×10^{7}	$H_3O^+ + OH^- \rightarrow H_2O$	3, 6	1.13×10^{11}
$H^\bullet + O_2 \rightarrow HO^\bullet_2$	2	1.27×10^{10}	$H_3O^+ + O^{\bullet-}_3 \rightarrow {}^\bullet OH + O_2$	3	9.00×10^{10}
$H^\bullet + HO^\bullet_2 \rightarrow H_2O_2$	2	1.00×10^{10}	$e^-_{aq} + H_3O^+ \rightarrow H^\bullet$	4, 6	2.11×10^{10}
$H^\bullet + O^{\bullet-}_2 \rightarrow HO^-_2$	2	1.00×10^{10}	$e^-_{aq} + O^{\bullet-}_2 \rightarrow H_2O_2 + 2OH^-$	4	1.28×10^{10}
${}^\bullet OH + {}^\bullet OH \rightarrow H_2O_2$	2	4.40×10^{9}	$e^-_{aq} + HO^-_2 \rightarrow O^{\bullet-} + OH^-$	4	3.51×10^{9}
${}^\bullet OH + H_2O_2 \rightarrow HO^\bullet_2 + H_2O$	2	2.88×10^{7}	$e^-_{aq} + O^{\bullet-} \rightarrow 2OH^-$	4	2.31×10^{10}
${}^\bullet OH + H_2 \rightarrow H^\bullet + H_2O$	2	4.17×10^{7}	$H_3O^+ + O^{\bullet-}_2 \rightarrow HO^\bullet_2$	4, 6	4.78×10^{10}
${}^\bullet OH + e^-_{aq} \rightarrow OH^-$	2	2.95×10^{10}	$H_3O^+ + HO^-_2 \rightarrow H_2O_2$	4, 6	4.78×10^{10}
${}^\bullet OH + OH^- \rightarrow O^{\bullet-} + H_2O$	2, 6	1.27×10^{10}	$H_3O^+ + O^{\bullet-} \rightarrow {}^\bullet OH$	4, 6	4.78×10^{10}
${}^\bullet OH + HO^\bullet_2 \rightarrow O_2 + H_2O$	2	9.79×10^{9}	$O^{\bullet-}_2 + O^{\bullet-} \rightarrow O_2 + 2OH^-$	4	6.00×10^{8}
${}^\bullet OH + O^{\bullet-}_2 \rightarrow O_2 + OH^-$	2	1.02×10^{10}	$HO^-_2 + O^{\bullet-} \rightarrow O^{\bullet-}_2 + OH^-$	4	3.50×10^{8}
${}^\bullet OH + HO^-_2 \rightarrow HO^\bullet_2 + OH^-$	2	8.32×10^{9}	$O^{\bullet-} + O^{\bullet-} \rightarrow H_2O_2 + 2OH^-$	4	9.00×10^{8}
${}^\bullet OH + O^{\bullet-} \rightarrow HO^-_2$	2	7.61×10^{9}	$O^{\bullet-} + O^{\bullet-}_3 \rightarrow 2O^{\bullet-}_2$	4	7.00×10^{8}
${}^\bullet OH + O^{\bullet-}_3 \rightarrow HO^\bullet_2 + O^{\bullet-}_2$	2	8.50×10^{9}	$HO^\bullet_2 \rightarrow H_3O^+ + O^{\bullet-}_2$	6	7.15×10^{5}*
$H_2O_2 + e^-_{aq} \rightarrow OH^- + {}^\bullet OH$	2	1.41×10^{10}	$O^{\bullet-}_3 \rightarrow O^{\bullet-} + O_2$	6	2.66×10^{3}*
$H_2O_2 + OH^- \rightarrow HO^-_2 + H_2O$	2, 6	1.27×10^{10}	$H^\bullet \rightarrow e^-_{aq} + H_3O^+$	6	5.94*
$H_2O_2 + O(^3P) \rightarrow HO^\bullet_2 + {}^\bullet OH$	2	1.27×10^{6}	$e^-_{aq} + H_2O \rightarrow H^\bullet + OH^-$	6	15.8
$H_2O_2 + O^{\bullet-} \rightarrow HO^\bullet_2 + OH^-$	2	5.55×10^{8}	$O^{\bullet-}_2 + H_2O \rightarrow HO^\bullet_2 + OH^-$	6	0.15
$H_2 + O(^3P) \rightarrow H^\bullet + {}^\bullet OH$	2	4.80×10^{3}	$HO^-_2 + H_2O \rightarrow H_2O_2 + OH^-$	6	1.36×10^{6}
$H_2 + O^{\bullet-} \rightarrow H^\bullet + OH^-$	2	1.21×10^{8}	$O(^3P) + H_2O \rightarrow 2\ {}^\bullet OH$	6	1.90×10^{3}
$e^-_{aq} + O_2 \rightarrow O^{\bullet-}_2$	2	1.84×10^{10}	$O^{\bullet-} + H_2O \rightarrow {}^\bullet OH + OH^-$	6	1.36×10^{6}

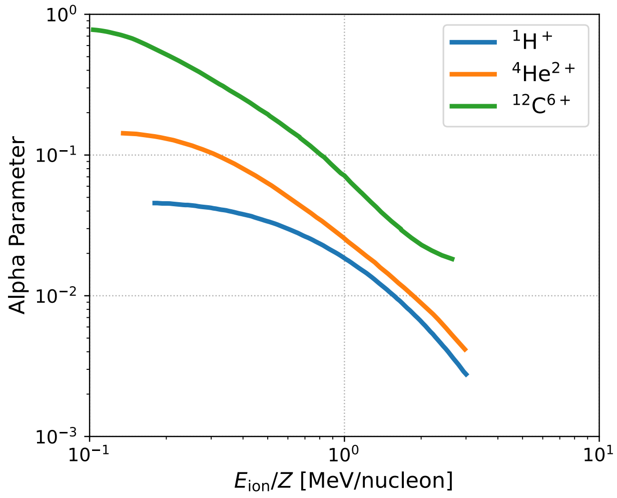

Figure S2. The scale parameter α for each projectile to scale the cross-section value of each multiple-ionization process. Detailed description of this parameter is in [22].

Table S4. The cross-section definition and energy threshold for each multiple-ionization process [22].

	Double Ionization	Triple Ionization	Quadruple Ionization
**Cross-section**	$\sigma_{di} = \alpha\sigma_{si}$	$\sigma_{ti} = \alpha^2\sigma_{si}$	$\sigma_{di} = \alpha^3\sigma_{si}$
**Energy Threshold**	40 eV	65 eV	88 eV

Table S5. Dissociative decay channels for multiple-ionized water molecules at the physico-chemical stage. The distances between fragments are given in nanometers [22,23].

**Double-ionized state: H_2O^{2+}**	→	$H^+ + H^+ + O(^3P)$	→	$2H_3O^+ + O(^3P)$	55%	$d_{O\text{-}H+} = 1.20$
	→	$H^+ + OH^+$	→	$2H_3O^+ + O(^3P)$	29%	$d_{H+\text{-}OH+} = 1.20$
	→	$H^+ + H^\bullet + O^+$	→	$2H_3O^+ + H^\bullet + {}^\bullet OH + O(^3P)$	16%	$d_{H+\text{-}O+} = 1.20$; $d_{H\bullet\text{-}O+} = 0.80$
**Triple-ionized state: H_2O^{3+}**	→	$H^+ + H^+ + O^+$	→	$3H_3O^+ + {}^\bullet OH + O(^3P)$	100%	$d_{O+\text{-}H+} = 1.20$
**Quadruple-ionized state: H_2O^{4+}**	→	$H^+ + H^+ + 2O^+$	→	$4H_3O^+ + 2{}^\bullet OH + O(^3P)$	100%	$d_{O+\text{-}H+} = 1.20$

S4 Fricke Dosimeter

For the validation of water radiolysis with a long timescale over several tens to hundreds of seconds, we performed a Fricke Dosimeter simulation using our GFDE-SBS model. This dosimeter consists of an aerated solution of ferrous sulfate ($FeSO_4$) and sulfuric acid (H_2SO_4) diluted in water. In this solution, the oxidation of Fe^{2+} and Fe^{3+} will be generated through the following chemical reactions:

$$
\begin{aligned}
&{}^{\cdot}\mathrm{OH} + \mathrm{Fe}^{2+} \rightarrow \mathrm{Fe}^{3+} + \mathrm{OH}^{-} \\
&\mathrm{H}^{\cdot} + \mathrm{O}_2 \rightarrow \mathrm{HO}_2^{\cdot} \\
&\mathrm{HO}_2^{\cdot} + \mathrm{Fe}^{2+} \rightarrow \mathrm{H}_2\mathrm{O}_2 + \mathrm{Fe}^{3+} \\
&\mathrm{H}_2\mathrm{O}_2 + \mathrm{Fe}^{2+} \rightarrow \mathrm{Fe}^{3+} + {}^{\cdot}\mathrm{OH} + \mathrm{OH}^{-}
\end{aligned}
\tag{14}
$$

From these reactions, we can find the well-known material equations (15), leading to $G(Fe^{3+})$ = 15.6 species/100eV [2]:

$$
\mathrm{G}(\mathrm{Fe}^{3+}) = \mathrm{G}({}^{\cdot}\mathrm{OH}) + 2\mathrm{G}(\mathrm{H}_2\mathrm{O}_2) + 3\mathrm{G}(\mathrm{H}^{\cdot}) \tag{15}
$$

The reaction rate constant for the Fenton reaction, which is the fourth in Equation (14), is 52 $(M\bullet s)^{-1}$, and the concentration of Fe^{3+} in a Fricke solution is 5 mM. The scavenging power of this reaction is derived to 0.26 s^{-1}. Thus, we need to simulate up to several ten seconds to complete this reaction. The chemical reactions involving $FeSO_4$ and H_2SO_4 were handled as background reactions. Therefore, we considered additional chemical reactions related to the Fricke solution listed in Table S6 and Table S7.

Table S6. Additional chemical reactions related to Fricke solution [1-4].

Reactions	Reaction rate constants [/(M•s)]
${}^{\cdot}OH + HSO_4^- \rightarrow H_2O + SO_4^-$	1.5×10^5
${}^{\cdot}H + SO_4^- \rightarrow HSO_4^-$	1.0×10^{10}
$H_2O_2 + SO_4^- \rightarrow HO_2^{\cdot} + HSO_4^-$	1.2×10^7
$OH^- + SO_4^- \rightarrow {}^{\cdot}OH + SO_4^{2-}$	8.3×10^7
$SO_4^- + SO_4^- \rightarrow S_2O_8^{2-}$	4.4×10^8
$SO_4^- + Fe^{2+} \rightarrow Fe^{3+} + SO_4^{2-}$	2.79×10^8
$e_{aq}^- + S_2O_8^{2-} \rightarrow SO_4^- + SO_4^{2-}$	1.2×10^{10}
${}^{\cdot}H + S_2O_8^{2-} \rightarrow SO_4^- + HSO_4^-$	2.5×10^7
$Fe^{2+} + {}^{\cdot}OH \rightarrow Fe^{3+} + OH^-$	3.4×10^8
$Fe^{2+} + HO_2^{\cdot} \rightarrow Fe^{3+} + HO_2^-$	7.9×10^5
$Fe^{2+} + H_2O_2 \rightarrow Fe^{3+} + {}^{\cdot}OH + OH^-$	52

Table S7. Additional molecular species for Fricke dosimeter simulations and diffusion coefficient for each referred from [1,5].

Species	Diffusion coefficient [m^2/s]	Species	Diffusion coefficient [m^2/s]
Fe^{2+}	5.78×10^{-10}	$SO_4^{\cdot-}$	1.385×10^{-9}
Fe^{3+}	4.86×10^{-10}	SO_4^{2-}	1.065×10^{-9}
HSO_4^-	1.385×10^{-9}	$S_2O_8^{2-}$	1.145×10^{-9}

S5 Comparison of the radiochemical yields calculated in CONV-SBS with ones by GFDE-SBS without considering electrostatic forces, background reactions, and spin effects

As described in the main manuscript, CONV-SBS cannot consider electrostatic forces amog charged molecules, background reactions involving dissolved species, and spin effects. Thus, we retried to assess the differences in radiochemical yields of each molecular species calculated from CONV-SBS and GFDE-SBS without the three effects. The differences in G values between the two models got closer, as shown in Figure S3. Still, depending on molecular species, specifically hydroxyl radicals and hydrogen peroxides, we observed some differences in G values, which could be caused by the differences in the reaction theory applied in each model: the combination of the Smoluchowski theory and the Brownian Bridge technique for CONV-SBS while the GFDE theory for GFDE-SBS.

S6 The impact induced by electrostatic forces, background reactions, and spin effects on radiochemical yields in GFDE-SBS

We have investigated the contribution of these three effects, including electrostatic forces among charged molecules, background reactions involving dissolved molecules, and spin effects, to the time evolution of radiochemical yields in GFDE-SBS, as shown in Figure S4. Here, we set the four cases. Case#0 is without considering the three effects. In Case#1, only electrostatic forces are applied. Case#2 adds background reactions to Case#1, while Case#3 considers all three effects. Regarding hydroxyl radicals, hydrated electrons, and hydrogen peroxides, we have not confirmed significant differences induced by each effect in their G values. For hydrogen radicals, the spin effect affects the approximately 30% increase in yields of hydrogen molecules. For OH^- anions, the effects of electrostatic forces among nearby charged molecular species increase their

yields by approximately 10%. Additionally, as described in the main document, the background reactions originating from H3O^{+} and OH^{-} produced by the self-dissociation of H_2O molecules impact the G values of OH^{-} anions. Its radiochemical yields decrease at approximately 200 nanoseconds later due to the chemical reactions with dissolved H_3O^{+}.

S7 LET calculations

For the validation of water radiolysis simulations with the GFDE-SBS approach under heavy ion irradiations, we compared the LET dependencies of the chemical yields calculated for molecular species with the experiment data reported. In the present work, LET for each projectile was calculated as a track-averaged LET_t [24]:

$$LET_t = \sum_{i=1}^{n} \left(\frac{\varepsilon_i}{l_i}\right) w_{i,t} = \frac{\sum_{i=1}^{n} \varepsilon_i}{\sum_{i=1}^{n} l_i}, \tag{16}$$

where ε_i is the energy deposition by i th charged particle with the step length l_i, and $w_{i,t} = l_i/\sum_{i=1}^{n} l_i$ is the track-length weighting factor the of i th event. LET_t is the ratio of the total energy depositions over the total step length for the primary particle. Following Eq. (16), we evaluated LET for each primary particle with varying incident energy. The results are shown in Figure S5. Our calculation results for protons, alpha particles, and carbon ions are solid lines with different colors. The other plots are computed by the SRIM code [25], which is widely used to obtain various types of physical quantities related to radiation physics. Our results are in good agreement with the SRIM outputs.

S8 Time evolution of the radiochemical yields under ion irradiation

Figure S6 shows the time evolution of radiochemical yields of hydrated electrons and hydroxyl radicals under ion irradiation, including $^{1}H^{+}$, $^{4}He^{2+}$, and $^{12}C^{6+}$, with an incident energy of 1, 2, 4, and 8 MeV/u. For each ion irradiation, the G value of each species rapidly decreases as LET increases. That is because, with LET increasing, primary species, like e_{aq}^{-} and $^{\bullet}OH$, are densely generated along the ion tracks, stimulating intratrack reactions.

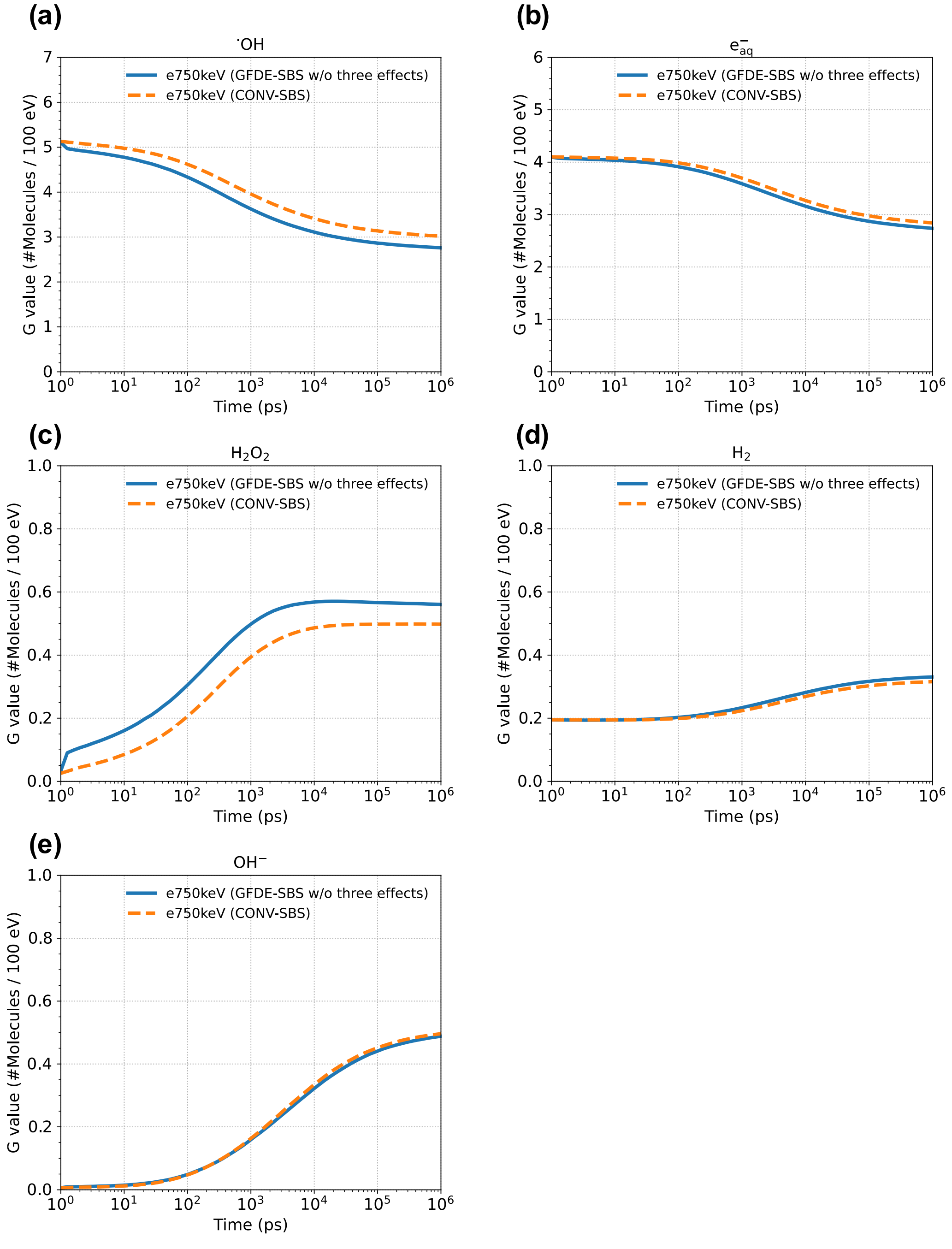

Figure S3. Comparison of the time evolution of G values calculated by GFDE-SBS (the blue solid lines) and CONV-SBS (the orange dashed lines) for (a) hydroxyl radicals, (b) hydrated electrons, (c) hydrogen peroxides, (d) hydrogen molecules, and (e) OH^- anions under 750 keV electron irradiation. Electrostatic forces among charged molecules, background reactions, and spin effects are not considered in GFDE-SBS.

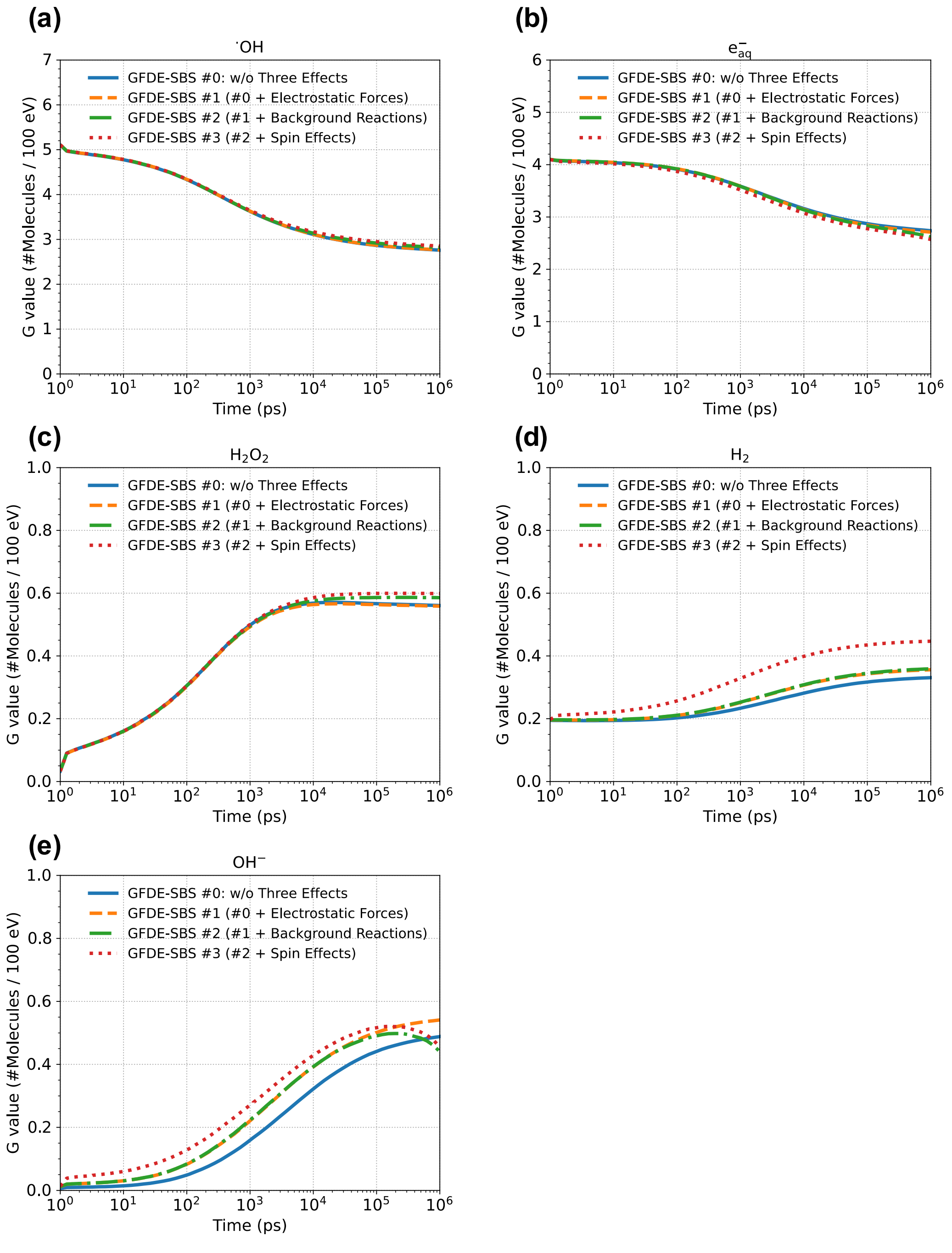

Figure S4. The time evolution of G values for (a) •OH, (b) e_{aq}^-, (c) H_2O_2, (d) H_2, and (e) OH^- under 750 keV electron irradiation. These were calculated in GFDE-SBS with the four cases: without the three effects (Case#0, the blue solid lines), considering with electrostatic forces (Case#1, the orange dashed lines), background reactions (Case#2, the green dash-dotted lines), and spin effects (Case#3, the red dotted lines).

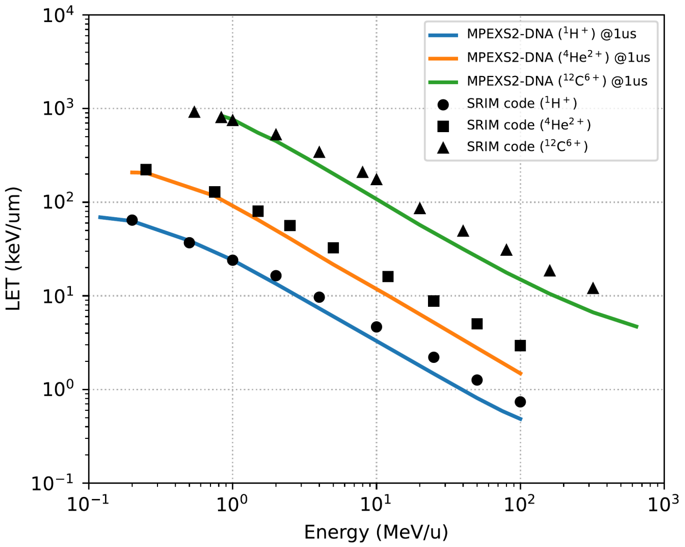

Figure S5. Comparison calculated LET between MPEXS2.1-DNA (solid lines with different colors) and the SRIM code.

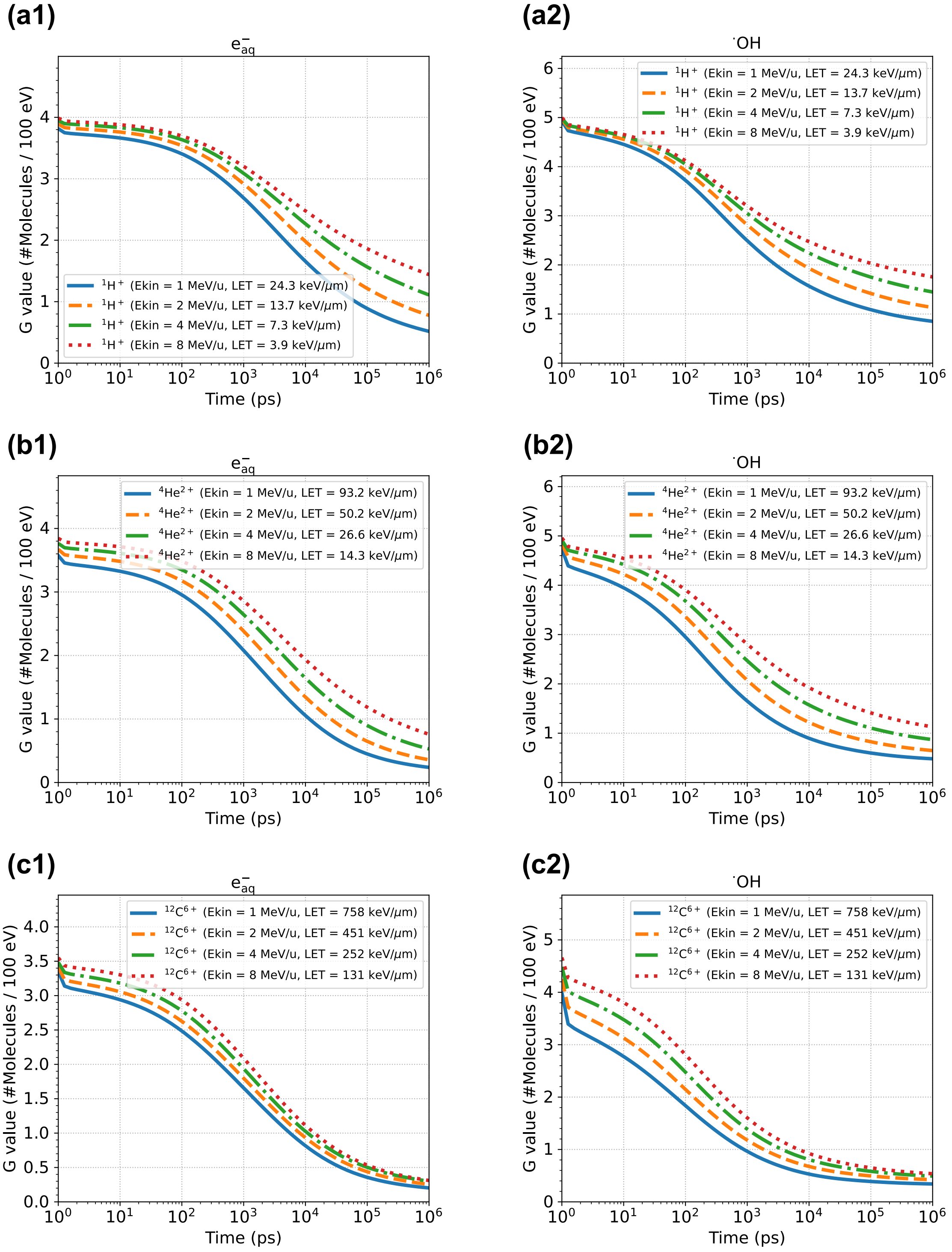

Figure S6. The time evolution of radiochemical yields of (1) hydrated electrons and (2) hydroxyl radicals under ion irradiation, (a) $^{1}H^{+}$, (b) $^{4}He^{2+}$, and (c) $^{12}C^{6+}$, with an incident energy of 1, 2, 4, and 8 MeV/u.

References

1 Autsavapromporn, N., Meesungnoen, J., Plante, I. & Jay-Gerin, J.-P. Monte Carlo simulation study of the effects of acidity and LET on the primary free-radical and molecular yields of water radiolysis — Application to the Fricke dosimeter. *Canadian Journal of Chemistry* **85**, 214-229, doi:10.1139/v07-021 (2007).

2 Plante, I. A Monte-Carlo step-by-step simulation code of the non-homogeneous chemistry of the radiolysis of water and aqueous solutions—Part II: calculation of radiolytic yields under different conditions of LET, pH, and temperature. *Radiation and Environmental Biophysics* **50**, 405-415, doi:10.1007/s00411-011-0368-7 (2011).

3 Meesat, R. *et al.* Utilization of the ferrous sulfate (Fricke) dosimeter for evaluating the radioprotective potential of cystamine: experiment and Monte Carlo simulation. *Radiat Res* **177**, 813-826, doi:10.1667/rr2829.1 (2012).

4 Ramos-Mendez, J. *et al.* LET-Dependent Intertrack Yields in Proton Irradiation at Ultra-High Dose Rates Relevant for FLASH Therapy. *Radiat Res* **194**, 351-362, doi:10.1667/RADE-20-00084.1 (2020).

5 Spiro, M. & Creeth, A. M. Tracer diffusion coefficients of I–, I, Fe2+ and Fe3+ at low temperatures. *Journal of the Chemical Society, Faraday Transactions* **86**, 3573-3576, doi:10.1039/FT9908603573 (1990).

6 Buxton, G. V. & Dainton, F. S. Nanosecond pulse radiolysis of aqueous solutions containing proton and hydroxyl radical scavengers. *Proceedings of the Royal Society of London. A. Mathematical and Physical Sciences* **328**, 9-21, doi:doi:10.1098/rspa.1972.0065 (1972).

7 Jonah, C. D., Hart, E. J. & Matheson, M. S. Yields and decay of the hydrated electron at times greater than 200 picoseconds. *The Journal of Physical Chemistry* **77**, 1838-1843, doi:10.1021/j100634a004 (1973).

8 Wolff, R. K., Bronskill, M. J., Aldrich, J. E. & Hunt, J. W. Picosecond pulse radiolysis. IV. Yield of the solvated electron at 30 picoseconds. *The Journal of Physical Chemistry* **77**, 1350-1355, doi:10.1021/j100630a008 (1973).

9 Draganić, Z. D. & Draganić, I. G. Formation of primary reducing yields (Geaq- and GH2) in the radiolysis of aqueous solutions of some positive ions. *International Journal for Radiation Physics and Chemistry* **7**, 381-386, doi:https://doi.org/10.1016/0020-7055(75)90077-7 (1975).

10 Jonah, C. D., Matheson, M. S., Miller, J. R. & Hart, E. J. Yield and decay of the hydrated electron from 100 ps to 3 ns. *The Journal of Physical Chemistry* **80**, 1267-1270, doi:10.1021/j100553a001 (1976).

11 Jonah, C. D. & Miller, J. R. Yield and Decay of the Hydroxyl Radical from 200ps to 3ns. *The Journal of Physical Chemistry* **81**, 1974-1976, doi:10.1021/j100536a002 (1977).

12 Sumiyoshi, T. & Katayama, M. THE YIELD OF HYDRATED ELECTRONS AT 30 PICOSECONDS. *Chemistry Letters* **11**, 1887-1890, doi:10.1246/cl.1982.1887 (1982).

13 Shiraishi, H., Katsumura, Y., Hiroishi, D., Ishigure, K. & Washio, M. Pulse-radiolysis study on the yield of hydrated electron at elevated temperatures. *The Journal of Physical Chemistry* **92**, 3011-3017, doi:10.1021/j100321a061 (1988).

14 A. LaVerne, J. OH Radicals and Oxidizing Products in the Gamma Radiolysis of Water. *Radiation Research* **153**, 196-200, doi:10.1667/0033-7587(2000)153[0196:ORAOPI]2.0.CO;2 (2000).

15 Muroya, Y. *et al.* A re-evaluation of the initial yield of the hydrated electron in the picosecond time range. *Radiation Physics and Chemistry* **72**, 169-172, doi:https://doi.org/10.1016/j.radphyschem.2004.09.011 (2005).

16 El Omar, A. K. *et al.* Time-Dependent Radiolytic Yield of OH• Radical Studied by Picosecond Pulse Radiolysis. *The Journal of Physical Chemistry A* **115**, 12212-12216, doi:10.1021/jp208075v (2011).

17 Pachnerová Brabcová, K. *et al.* Contribution of indirect effects to clustered damage in DNA irradiated with protons. *Radiation Protection Dosimetry* **166**, 44-48, doi:10.1093/rpd/ncv159 (2015).

18 Plante, I. A Monte–Carlo step-by-step simulation code of the non-homogeneous chemistry of the radiolysis of water and aqueous solutions. Part I: theoretical framework and implementation. *Radiation and Environmental Biophysics* **50**, 389-403, doi:10.1007/s00411-011-0367-8 (2011).

19 Plante, I. & Devroye, L. Considerations for the independent reaction times and step-by-step methods for radiation chemistry simulations. *Radiation Physics and Chemistry* **139**, 157-172, doi:10.1016/j.radphyschem.2017.03.021 (2017).

20 Plante, I. A review of simulation codes and approaches for radiation chemistry. *Physics in Medicine & Biology* **66**, 03TR02, doi:10.1088/1361-6560/abbd19 (2021).

21 Frongillo, Y. *et al. Monte Carlo simulation of fast electron and proton tracks in liquid water - II. Nonhomogeneous chemistry*. Vol. 51 (1998).

22 Meesungnoen, J. & Jay-Gerin, J.-P. High-LET Radiolysis of Liquid Water with 1H+, 4He2+, 12C6+, and 20Ne9+ Ions: Effects of Multiple Ionization. *The Journal of Physical Chemistry A* **109**, 6406-6419, doi:10.1021/jp058037z (2005).

23 Gervais, B., Beuve, M., Olivera, G. H. & Galassi, M. E. Numerical simulation of multiple ionization and high LET effects in liquid water radiolysis. *Radiation Physics and Chemistry* **75**, 493-513, doi:10.1016/j.radphyschem.2005.09.015 (2006).

24 Shin, W. G. *et al.* Evaluation of the influence of physical and chemical parameters on water radiolysis simulations under MeV electron irradiation using Geant4-DNA. *Journal of Applied Physics* **126**, 114301, doi:10.1063/1.5107511 (2019).

25 Ziegler, J. F., Ziegler, M. D. & Biersack, J. P. SRIM – The stopping and range of ions in matter (2010). *Nuclear Instruments and Methods in Physics Research Section B: Beam Interactions with Materials and Atoms* **268**, 1818-1823, doi:10.1016/j.nimb.2010.02.091 (2010).
